# Idealized Computational Models for Auditory Receptive Fields

**DOI:** 10.1371/journal.pone.0119032

**Published:** 2015-03-30

**Authors:** Tony Lindeberg, Anders Friberg

**Affiliations:** 1 Department of Computational Biology, School of Computer Science and Communication, KTH Royal Institute of Technology, Stockholm, Sweden; 2 Department of Speech, Music and Hearing, School of Computer Science and Communication, KTH Royal Institute of Technology, Stockholm, Sweden; Max Planck Institute for Human Cognitive and Brain Sciences, GERMANY

## Abstract

We present a theory by which idealized models of auditory receptive fields can be derived in a principled axiomatic manner, from a set of structural properties to (i) enable invariance of receptive field responses under natural sound transformations and (ii) ensure internal consistency between spectro-temporal receptive fields at different temporal and spectral scales. For defining a time-frequency transformation of a purely temporal sound signal, it is shown that the framework allows for a new way of deriving the Gabor and Gammatone filters as well as a novel family of generalized Gammatone filters, with additional degrees of freedom to obtain different trade-offs between the spectral selectivity and the temporal delay of time-causal temporal window functions. When applied to the definition of a second-layer of receptive fields from a spectrogram, it is shown that the framework leads to two canonical families of spectro-temporal receptive fields, in terms of spectro-temporal derivatives of either spectro-temporal Gaussian kernels for non-causal time or a cascade of time-causal first-order integrators over the temporal domain and a Gaussian filter over the logspectral domain. For each filter family, the spectro-temporal receptive fields can be either separable over the time-frequency domain or be adapted to local glissando transformations that represent variations in logarithmic frequencies over time. Within each domain of either non-causal or time-causal time, these receptive field families are derived by uniqueness from the assumptions. It is demonstrated how the presented framework allows for computation of basic auditory features for audio processing and that it leads to predictions about auditory receptive fields with good qualitative similarity to biological receptive fields measured in the inferior colliculus (ICC) and primary auditory cortex (A1) of mammals.

## Introduction

The information in sound is based on variations in the air pressure over time, which for many sound sources can be modelled as a superposition of sine wave oscillations of different frequencies. To capture this information by auditory perception or signal processing, the sound signal has to be processed over some non-infinitesimal amount of time and in the case of a spectral analysis also over some range of frequencies. Such a region over time or over the spectro-temporal domain is referred to as a temporal or spectro-temporal *receptive field* (Aertsen and Johannesma [1]; Theunissen et al. [2]; Miller et al. [3]; Fritz et al. [4]).

If one considers the theoretical or algorithmic problem of designing an auditory system that is going to analyse the variations in air pressure over time, one may ask what types of auditory operations should be performed on the sound signal. Would any operation be reasonable? Specifically, regarding the notion of receptive fields, what types of temporal or spectro-temporal receptive field profiles would be reasonable? Is it possible to derive a theoretical model of how receptive fields “ought to” respond to auditory signals?

In vision, the corresponding problem of formulating a theoretical model for visual receptive fields (Lindeberg [5]) can be addressed based on a framework developed in the area of computer vision known as *scale-space theory* (Iijima [6]; Witkin [7]; Koenderink [8]; Koenderink and van Doorn [9, 10]; Lindeberg [11–14]; Sporring et al. [15]; Florack [16]; ter Haar Romeny [17]). A paradigm that has been developed in this field is to impose *structural constraints* on the first stages of processing that reflect *symmetry properties* of the environment. Interestingly, it turns out to be possible to substantially reduce the class of permissible image operations from such arguments, and it has been shown that biological receptive fields as measured in the lateral geniculate nucleus (LGN) and the primary visual cortex (V1) of higher mammals (Hubel and Wiesel [18–20]; DeAngelis et al. [21, 22]; Conway and Livingstone [23]; Johnson et al. [24]) can be well modelled by idealized scale-space operations (Young et al. [25, 26]; Lindeberg [5, 27]).

The subject of this article is to show how a corresponding normative theory for receptive fields can be developed for auditory stimuli, and how idealized models of auditory receptive fields can be derived in a principled manner by applying scale-space theory to auditory signals. Our aim is to express auditory operations that are well localized over time and frequencies and which allow for well-founded handling of temporal phenomena that occur at different temporal scales as well as receptive fields that operate over different ranges of frequencies in such a way that operations over different ranges of frequencies can be related in a well-defined manner.

When applied to the definition of spectrograms, alternatively to the formulation of an idealized cochlea model, the scale-space approach can be used for deriving the Gabor (Gabor [28]; Wolfe et al. [29]; Lobo and Loizou [30]; Qiu et al. [31]; Wu et al. [32]) and Gamma-tone (Johannesma [33]; Patterson et al. [34]; Hewitt and Meddis [35, 36]) approaches for computing local windowed Fourier transforms as specific cases of a complex-valued scale-space transform over different frequencies. In addition, the scale-space approach to defining spectrograms leads to a new family of *generalized Gamma-tone filters*, where the time constants of the individual first-order integrators coupled in cascade are not equal as for regular Gamma-tone filters but instead distributed logarithmically over temporal scales and allowing for different trade-offs in terms of *e.g*. the frequency selectivity of the spectrogram and the temporal delay of time-causal receptive fields.

When applied to a logarithmic transformation of the spectrogram, as motivated from the desire of handling sound signals of different strength (sound pressure) in an invariant manner and with a logarithmic transformation of the frequencies as motivated by the desire of enabling invariance properties under a frequency shift, such as transposing a musical piece by one octave, we will show how this theory also allows for the formulation of spectro-temporal receptive fields at higher levels in the auditory hierarchy in terms of spectro-temporal derivatives of spectro-temporal smoothing operations as obtained from scale-space theory.

It will be demonstrated how such second-layer receptive fields can be used for *computing basic auditory features* such as onset detection, partial tone enhancement and formants, and specifically how different types of features can be obtained at different temporal scales *τ*, spectral scales *s* and how this theory naturally also leads to a glissando parameter *v* that represents how logarithmic frequencies *ν* may vary over time *t* according to a local linear approximation *ν*′ = *ν* + *vt*.

Compared to the more common approach of computing auditory features in digital signal processing by local windowed fast Fourier transforms (FFT), we argue that the proposed theory provides a way to avoid artifacts of performing the computation in temporal blocks that later have to be combined again. Furthermore, by the built-in covariance properties of the model under temporal shifts, variations in sound pressure, frequency shifts and glissando transformations, the proposed approach allows for *provable invariance properties* under such transformations of sound signals.

It will also be shown how idealized models of spectro-temporal receptive fields as obtained from the presented theory in terms of spectro-temporal derivatives of spectro-temporal scale-space kernels can be used for generating *predictions of auditory receptive fields that are qualitatively similar to biological receptive fields* as measured by cell recordings in the inferior colliculus (ICC) and the primary auditory cortex (A1) (Miller et al. [3]; Qiu et al. [31]; Machens et al. [37]; Andoni et al. [38]; Elhilali et al. [39]; Atencio and Schreiner [40]).

### Outline of the presentation

The presentation is organized as follows: The section “Structural requirements on temporal receptive fields” describes basic constraints on temporal receptive fields as motivated by the desire of capturing temporal structures at different temporal scales in a theoretically well-defined manner. The section “Scale-space concepts for purely temporal domains” then describes the temporal scale-space concepts that satisfy these properties, with a distinction on whether the auditory processing operations are required to be time-causal or not. For off-line processing of pre-recorded sound signals, we may take the liberty of accessing the virtual future in relation to any pre-recorded time moment, whereas one in a real-time situation has to take the fact that the future cannot be accessed into explicit account. Thereby, we obtain different theories depending on whether time is treated in a non-causal or a time-causal manner.

In the section “Multi-scale spectrograms for auditory signals” we apply these temporal scale-space theories to the definition of multi-scale spectrograms by the formulation of locally windowed Fourier transforms of different temporal extent to be able to capture temporal phenomena at different temporal scales. The section “Receptive fields defined over the spectrogram” develops a corresponding theory for spectro-temporal receptive fields applied to the spectrogram, and it is shown how auditory receptive fields over the spectro-temporal domain can be expressed in an analogous way to how visual receptive fields are defined over space-time, with the conceptual difference that the two spatial dimensions in vision are replaced by a logarithmic frequency dimension. Specifically, we demonstrate how basic auditory features can be computed in this way from spectro-temporal derivatives of idealized receptive fields as obtained from the auditory scale-space theory.

The section “Relations to biological receptive fields” gives examples of how biological auditory receptive fields can be modelled by the proposed theory. The section “Relations to previous work in audio processing” relates the presented theory to previous approaches in audio processing, and the section “Summary and discussion” concludes with an overall summary of the contributions in the paper, implications of the theory and directions for future work.

The section “Frequency selectivity of the spectrograms” complements the above treatment by an in-depth analysis of the frequency selectivity properties of the temporal scale-space kernels. The section “Temporal dynamics of the time-causal kernels” gives a corresponding analysis of the temporal delays of the time-causal receptive fields. Finally, the section “Computational implementation” shows how the presented continuous theory can be transferred to a discrete implementation while still preserving the theoretical scale-space properties, and thereby allowing for theoretically well-founded digital implementation *e.g*. for digital audio signal processing or computational modelling of auditory perception.

## Structural requirements on temporal receptive fields

In the following, we will describe a set of structural requirements concerning temporal receptive fields for a general sensory system that processes a scalar time-dependent signal regarding (i) the measurement of sensory data with its close relationship to the notion of temporal scale, (ii) internal derived representations of the signal that are to be computed by a general sensory system, and (iii) the special nature of time in terms of temporal causality and temporal recursivity.

If we regard the sensory signal *f* as defined on a one-dimensional continuous temporal axis *f* : ℝ → ℝ, then the problem of defining a set of early sensory operations can be formulated as finding a family of operators 𝓣_*τ*_ that are to act on *f* to produce a family of new intermediate representations of the signal

$$\begin{array}{c} L\left(\cdot ;\;\tau \right)={𝓣}_{\tau }\;f\left(\cdot \right) \end{array}$$ (1)

which are also to be defined as functions on ℝ, *i.e*., *L*(⋅; *τ*) : ℝ → ℝ.

(In Equation (1), the symbol “⋅” at the position of the first argument of *L* is a place holder to emphasize that in this relation, *L* is regarded as a function and not evaluated with respect to its first argument *t*. The following semi-colon emphasizes the different natures of the temporal coordinate *t* and the filter parameter *τ*.)

The evaluation of one (specific example) function *L* can be interpreted as the response of a (set of) sensory neurons in biology or as an internal representation in a temporal signal processing system that processes temporal information. Combined with additional processing over frequencies, we will later use such internal representations for modelling neurons in the inferior colliculus (ICC) and the primary auditory cortex (A1).

### General scale-space axioms for temporal receptive fields

#### Linearity

If we want the initial auditory processing stages to make as few irreversible decisions as possible, it is natural to require 𝓣_*τ*_ to be *linear* such that

$$\begin{array}{c} {𝓣}_{\tau }\left(a_{1}f_{1}+a_{2}f_{2}\right)=a_{1}{𝓣}_{\tau }f_{1}+a_{2}{𝓣}_{\tau }f_{2} \end{array}$$ (2)

holds for all functions *f*_1_, *f*_2_ : ℝ → ℝ and all scalar constants *a*_1_, *a*_2_ ∈ ℝ. The motivation for avoiding early irreversible decisions is that we would as much as possible like to preserve an isomorphic mapping to the input, not losing important information.

Linearity also implies that a number of special properties of receptive fields (to be developed below) transfer to temporal derivatives of these and do therefore imply that different types of time-dependent structures in the signal will be treated in a similar manner irrespective of what types of linear filters they are captured by.

Concerning the assumption of linearity, it should be noted that there is an implicit degree of freedom in this formulation concerning the parameterization of the units by which the input signal *f* is measured. Given an underlying measurement signal *I*(*t*) in units of energy from a sensor, one could for a positive signal also consider defining the input signal *f* in terms of a reparameterization of the sensor signal *I* according to a self-similar power law for some *α* > 0

$$\begin{array}{c} f\left(t\right)={\left(I(t)\right)}^{\alpha } \end{array}$$ (3)

or a self-similar logarithmic transformation

$$\begin{array}{c} f\left(t\right)=\log\left(\frac{I(t)}{I_{0}}\right) \end{array}$$ (4)

defined relative to some reference level *I*_0_. Both of these transformations are self-similar in the sense that (i) they are well-behaved under rescalings of the measurement domain *I*(*t*) ↦ *a* *I*(*t*) for *a* > 0 and (ii) the local magnification/compression around any measurement value as defined from the derivative also follows a self-similar power law.

#### Temporal shift invariance

Let us require 𝓣_*τ*_ to be a *shift-invariant operator* in the sense that it commutes with the temporal shift operator 𝓢_Δ*t*_ defined by (𝓢_Δ*t*_ *f*)(*t*) = *f*(*t* − Δ*t*), such that

$$\begin{array}{c} {𝓣}_{\tau }\left({𝓢}_{\Delta t}f\right)={𝓢}_{\Delta t}\left({𝓣}_{\tau }f\right) \end{array}$$ (5)

holds for all Δ*t* ∈ ℝ. The motivation behind this assumption is the basic requirement that the representation of a sensory event should be similar irrespective of when it occurs.

#### Convolution structure

Together, the assumptions of linearity and shift-invariance imply that the internal representations *L*(⋅; *τ*) are given by *convolution transformations* (Hirschmann and Widder [41])

$$\begin{array}{c} L\left(t;\;\tau \right)=\left(T\left(\cdot ;\;\tau \right)*f\right)\left(t\right)=\int_{\xi \in \mathbb{R}}T\left(\xi ;\;\tau \right)\;f\left(t-\xi \right)\;d\xi \end{array}$$ (6)

where *T*(⋅; *τ*) denotes some family of convolution kernels. These kernels and their temporal derivatives can also be referred to as temporal receptive fields.

#### Regularity

To be able to use tools from functional analysis, we will initially assume that both the original signal *f* and the family of convolution kernels *T*(⋅; *τ*) are in the Banach space *L* ^2^(ℝ), *i.e*. that *f* ∈ *L* ^2^(ℝ) and *T*(⋅; *τ*) ∈ *L* ^2^(ℝ) with the norm

$$\begin{array}{c} {∥f∥}_{2}^{2}=\int_{t\in \mathbb{R}}{\left|f\left(t\right)\right|}^{2}\;dt. \end{array}$$ (7)

Then, also the intermediate representations *L*(⋅; *τ*) will be in the same Banach space, and the operators 𝓣_*τ*_ can be regarded as well-defined.

#### Positivity (non-negativity)

Concerning the convolution kernels *T*, one may require these to be non-negative to constitute smoothing transformations

$$\begin{array}{c} T(t;\;\tau )\geq 0. \end{array}$$ (8)

#### Normalization

Furthermore, it is natural to require the convolution kernels to be normalized to unit mass

$$\begin{array}{c} {∥T\left(\cdot ;\;\tau \right)∥}_{1}=\int_{t\in \mathbb{R}}T\left(t;\;\tau \right)\;dt=1 \end{array}$$ (9)

to leave a constant signal unaffected by the temporal smoothing transformation.

#### Quantitative measurement of the temporal extent and the temporal offset of non-negative scale-space kernels

For a non-negative convolution kernel, we can measure the temporal offset $m=\bar{t}$ by the temporal mean operator

$$\begin{array}{c} m=\bar{t}=M\left(T\left(\cdot ;\;\tau \right)\right)=\frac{\int_{t\in \mathbb{R}}t\;T\left(t;\;\tau \right)\;dt}{\int_{t\in \mathbb{R}}T\left(t;\;\tau \right)\;dt} \end{array}$$ (10)

and the temporal extent by the temporal variance

$$\begin{array}{c} \Sigma =V\left(T\left(\cdot ;\;\tau \right)\right)=\frac{\int_{t\in \mathbb{R}}{\left(t-\bar{t}\right)}^{2}\;T\left(t;\;\tau \right)\;dt}{\int_{t\in \mathbb{R}}T\left(t;\;\tau \right)\;dt}. \end{array}$$ (11)

Using the additive properties of mean values and variances under convolution, which hold for non-negative distributions, it follows that

$$\begin{array}{ccc} m & = & M\left(T\left(\cdot ;\;\tau_{1}\right)*T\left(\cdot ;\;\tau_{2}\right)\right)=M\left(T\left(\cdot ;\;\tau_{1}\right)\right)+M\left(T\left(\cdot ;\;\tau_{2}\right)\right)=m_{1}+m_{2}, \end{array}$$ (12)

$$\begin{array}{ccc} \Sigma & = & V\left(T\left(\cdot ;\;\tau_{1}\right)*T\left(\cdot ;\;\tau_{2}\right)\right)=V\left(T\left(\cdot ;\;\tau_{1}\right)\right)+V\left(T\left(\cdot ;\;\tau_{2}\right)\right)=\Sigma_{1}+\Sigma_{2}. \end{array}$$ (13)

#### Identity operation with continuity

To guarantee that the limit case of the internal scale-space representations when the scale parameter *τ* tends to zero should correspond to the original sound signal *f*, we will assume that

$$\begin{array}{c} \lim_{\tau ↓0}L\left(\cdot ;\;\tau \right)=\lim_{\tau ↓0}{𝓣}_{\tau }f=f. \end{array}$$ (14)

Hence, the intermediate signal representations *L*(⋅; *τ*) can be regarded as a family of derived representations parameterized by a temporal scale parameter *τ*.

#### Semi-group alternatively Markov structure over scale

For such sensory measurements to be properly related *between* different temporal scales, it is natural to require the operators 𝓣_*τ*_ with their associated convolution kernels *T*(⋅; *τ*) to form a *semi-group* over *τ*

$$\begin{array}{c} {𝓣}_{\tau_{1}}{𝓣}_{\tau_{2}}={𝓣}_{\tau_{1}+\tau_{2}} \end{array}$$ (15)

which means that the composition of two kernels from the semi-group should also be a member of the same family of kernels and with added parameter values

$$\begin{array}{c} T\left(\cdot ;\;\tau_{1}\right)*T\left(\cdot ;\;\tau_{2}\right)=T\left(\cdot ;\;\tau_{1}+\tau_{2}\right). \end{array}$$ (16)

Then, the transformation between any different and ordered scale levels *τ*_1_ and *τ*_2_ with *τ*_2_ ≥ *τ*_1_ will obey the *cascade property*

$$\begin{array}{ccc} L(\cdot ;\;\tau_{2}) & = & T\left(\cdot ;\;\tau_{2}-\tau_{1}\right)*T\left(\cdot ;\;\tau_{1}\right)*f=T\left(\cdot ;\;\tau_{2}-\tau_{1}\right)*L\left(\cdot ;\;\tau_{1}\right) \end{array}$$ (17)

implying that we can compute the representation *L*(⋅; *τ*_2_) at a coarser scale from the representation *L*(⋅; *τ*_1_) at any finer scale using a similar type of transformation as when computing the representation at any scale from the original data *f*.

For a temporal scale-space representation based on a discrete set of temporal scale levels *τ*_*k*_ (*k* = 0 … *K*), we can alternatively require a *Markov property* of the form

$$\begin{array}{c} T\left(\cdot ;\;\tau_{k+1}\right)=\left(\Delta T\right)\left(\cdot ;\;k\right)*T\left(\cdot ;\;\tau_{k}\right) \end{array}$$ (18)

where (Δ*T*)(⋅; *k*) represents the transformation between adjacent scale levels *τ*_*k*_ and *τ*_*ki*+1_. Then, the mapping between any pair of temporal scale levels *m* < *n*

$$\begin{array}{c} T\left(\cdot ;\;\tau_{n}\right)=\left(\Delta T\right)\left(\cdot ;\;m\mapsto n\right)*T\left(\cdot ;\;\tau_{m}\right) \end{array}$$ (19)

will be given by convolution with the kernel

$$\begin{array}{c} \left(\Delta T\right)\left(\cdot ;\;m\mapsto n\right)={*}_{k=m}^{n-1}\left(\Delta T\right)\left(\cdot ;\;k\right). \end{array}$$ (20)

The difference between the semi-group and the Markov assumptions is that the semi-group structure forces the transformations between adjacent scales to be independent of the current scale level, whereas the transformations between adjacent scales may vary with scale under the Markov assumption. The reason for relaxing the semi-group structure to a Markov structure is to make it possible to take larger temporal scale steps Δ*τ*_*k*_ at coarser temporal scales, which will have important implications for time-causal receptive fields.

A representation of a signal possessing these properties is called a *temporal multi-scale representation*.

#### Self-similarity over scale

Regarding the family of convolution kernels used for computing a multi-scale representation, one may require them to be *self-similar over temporal scales*, such that all the kernels correspond to rescaled copies

$$\begin{array}{c} T\left(t;\;\tau \right)=\frac{1}{\varphi (\tau )}\bar{T}\left(\frac{t}{\varphi (\tau )}\right) \end{array}$$ (21)

of some prototype kernel $\bar{T}$ for some transformation of *φ*(*τ*) of the temporal scale parameter *τ*.

The reason for introducing a function *φ* for transforming the scale parameter *s* into a scaling factor *φ*(*τ*) over time, is that the semi-group requirement (15) does not imply any restriction on how the parameter *τ* should be related to sound measurements in dimensions of time—the semi-group structure only implies an abstract ordering relation between coarser and finer scales *τ*_2_ > *τ*_1_ that could also be satisfied for any monotonously increasing transformation of the scale parameter *τ*. For the Gaussian temporal scale-space concept (25)–(26) this transformation is given by $\sigma =\varphi (\tau )=\sqrt{\tau }$.

#### Temporal covariance

If the same sensory stimulus is recorded by two sensors that sample the variations in the stimulus with different temporal sampling rates, or if similar temporal events occur at a somewhat different speed, it seems natural that the auditory system should be able to relate the temporal scale-space representations that are computed from the data.

Therefore, one may require that if the temporal dimension is rescaled by a uniform scaling factor *f*′ = 𝓢*f* corresponding to *f*′(*t*′) = *f*(*t*) with *t*′ = *S* *t*, then there should exist some transformation of the temporal scale *τ*′ = *B*(*τ*) such that the corresponding temporal scale-space representations are equal: *L*′(*t*′; *τ*′) = *L*(*t*; *τ*) corresponding to 𝓣_*B*(*τ*)_ℬ*f* = ℬ𝓣_*τ*_ *f*. In the case of a discrete set of temporal scale levels, we cannot however require self-similarity or temporal covariance to hold exactly. At best, we can aim at approximate transformation properties *e.g*. in terms of the temporal variance of the temporal scale-space kernels.

#### Non-creation of new structures with increasing scale

A necessary requirement on a scale-space representation is that convolution with the scale-space kernel *T*(⋅; *τ*) should correspond to a *smoothing transformation* in the sense that coarser scale representations should be guaranteed to constitute *simplifications* of corresponding finer scale representations, so that new structures are not created from finer to coarser scales:

#### Non-creation of local extrema (zero-crossings)

One way of formalizing such a requirement for a one-dimensional signal *f* : ℝ → ℝ, is by the requirement that the number of local extrema in the data must not increase with scale for any signal and is referred to as *non-creation of local extrema*. Formally, a one-dimensional kernel *T* is a scale-space kernel if for any signal *f*, the number of local extrema in *T* * *f* is guaranteed to not exceed the number of local extrema in *f* (Lindeberg [42]). For a one-dimensional signal, this condition can also be equivalently expressed in terms of zero-crossings.

### Specific scale-space axioms for a non-causal temporal domain

Depending on the conditions under which the sensory data is processed, we can consider two types of cases. For pre-recorded signals, we may in principle assume access to the data at all temporal moments simultaneously and thereby apply operations to the signal that would correspond to access to virtual future. For real-time signal processing or when modelling biological perception, there is, however, no way of having access to the future, which imposes fundamental additional structural requirements on a temporal front-end. For pre-recorded temporal signals, we require the following:

#### Non-enhancement of local extrema

**Fig 1:**
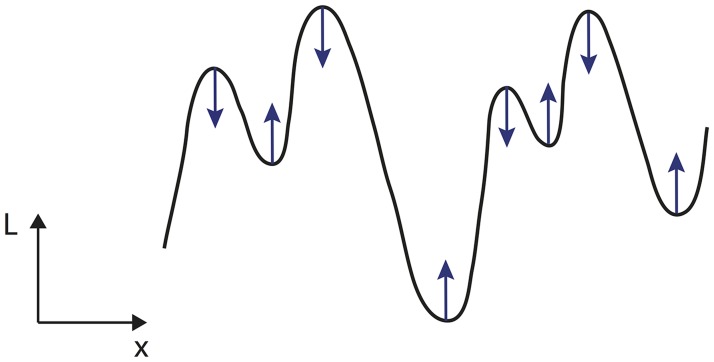
Illustration of the notion of non-enhancement of local extrema for a 1-D signal. The requirement of non-enhancement of local extrema is a way of restricting the class of possible filtering operations by formalizing the notion that new structures in the signal must not be created with increasing scale, by requiring that the value at a local maximum must not increase with scale and that the value at a local minimum must not decrease. (Figure from Lindeberg [27].)

In the case of a continuous scale parameter, one way of formalizing the requirement of non-creation of new structures in the signal with increasing scale is that *local extrema must not be enhanced with increasing scale*. In other words, if a point (*t*_0_; *τ*_0_) is a local (spatial) maximum of the mapping *t* ↦ *L*(*t*; *τ*_0_) then the value must not increase with scale. Similarly, if a point (*t*; *τ*_0_) is a local (spatial) minimum of the mapping *t* ↦ *L*(*t*; *τ*_0_), then the value must not decrease with scale (see fig. 1). Given the above mentioned differentiability property with respect to scale, we say that the multi-scale representation constitutes a *scale-space representation* if for a scalar scale parameter it satisfies the following conditions (Lindeberg [14, 43]):

$$\begin{array}{c} \partial_{\tau }L\left(t_{0};\;\tau_{0}\right)\leq 0\;\;\text{at}\;\text{any}\;\text{non-degenerate}\;\text{local}\;\text{maximum}, \end{array}$$ (22)

$$\begin{array}{c} \partial_{\tau }L\left(t_{0};\;t\tau_{0}\right)\geq 0\;\;\text{at}\;\text{any}\;\text{non-degenerate}\;\text{local}\;\text{minimum}. \end{array}$$ (23)

By considering the response to a constant signal, it follows from the requirement of non-enhancement of local extrema that a scale-space kernel should be normalized to unit *L*_1_-norm, corresponding to the normalization requirement in Equation (9).

### Specific scale-space axioms for a time-causal temporal domain

When processing sensory data in a real-time scenario, the following additional temporal requirements are instead needed:

#### Temporal causality

For a sensory system that interacts with the environment in a real-time setting, a fundamental constraint on the convolution kernels (the temporal receptive fields) is that there is no way of having access to future information, implying that the temporal smoothing kernels must be *time-causal* such that the convolution kernel must be zero for any relative time moment that would imply access to the future:

$$\begin{array}{c} T(t;\;\tau )=0\;\text{if}\;t<0. \end{array}$$ (24)

The possibly pragmatic solution of using a truncated symmetric filter of finite support in combination with a temporal delay may not be appropriate for a time-critical real-time system, since it would lead to unnecessarily long time delays in particular at coarser temporal scales. Therefore, a dedicated theory for truly time-causal spatio-temporal scale-space concepts is needed.

#### Time-recursivity

Another fundamental constraint on a real-time system is that it cannot be expected to keep a full record of everything that has happened in the past. To keep down memory requirements, it is desirable that the computations can be based on a limited internal *temporal buffer* *M*(*t*), which should provide:

- a sufficient record of past information and
- sufficient information to update its internal state in a recursive manner over time as new information arrives.

A particularly useful solution in this context is to use the internal temporal representations *L* at different temporal scales as a sufficient temporal memory buffer of the past.

#### Non-creation of structure in the context of discrete temporal scale levels

For a temporal scale-space representation involving a discrete set of scale levels only, we build on the requirement of non-creation of local extrema as expressed for a one-dimensional temporal signal depending on time *t* only. Let us therefore regard a one-dimensional temporal smoothing kernel *T*_*time*_ as a *temporal scale-space kernel* if and only if the kernel is (i) time-causal and in addition (ii) for any purely temporal signal *f*, the number of local extrema in *T*_*time*_ * *f* is guaranteed to not exceed the number of local extrema in *f* (Lindeberg and Fagerström [44]).

## Scale-space concepts for purely temporal domains

In this section we will describe how the structural requirements listed in the section “Structural requirements on temporal receptive fields” restrict the class of temporal scale-space kernels and thus the class of possible temporal receptive fields.

### Non-causal Gaussian temporal scale-space

If, for the purpose of analyzing pre-recorded auditory data, we allow for unlimited freedom of accessing the sensory data at all temporal moments simultaneously, we can apply a similar way of reasoning as has been used for deriving scale-space concepts for image data over a spatial domain (Iijima [6]; Witkin [7]; Koenderink [8]; Lindeberg [11–14, 43]; Sporring et al. [15]; Florack [16]; Weickert et al. [45]; ter Haar Romeny [17]):

Given time-dependent sensory data *f* : ℝ → ℝ defined over a one-dimensional temporal domain, let us assume that the first stage of sensory processing as represented by the operator 𝓣_*τ*_ should satisfy the following structural requirements: (i) *linearity*, (ii) *shift invariance* and (iii) obey a *semi-group structure over temporal scales* *τ*, where we also have to assume (iv) certain *regularity properties* of the semi-group 𝓣_*τ*_ *over scale* *τ* to guarantee sufficient differentiability properties with respect to time *t* and temporal scales *τ*. Let us furthermore require (iv) *non-enhancement of local extrema* to hold for *any* smooth function *f* ∈ *C* ^∞^(ℝ) ∩ *L* ^1^(ℝ) and for any positive scale direction *s*.

Then, it follows from (Lindeberg [14], theorem 5) that these conditions together imply that the scale-space family *L* must satisfy a diffusion equation of the form

$$\begin{array}{c} \partial_{\tau }L=\frac{1}{2}\;\Sigma_{0}\;\partial_{tt}L-\delta_{0}\;\partial_{t}L \end{array}$$ (25)

with initial condition *L*(*t*; 0) = *f*(*t*) for some positive constant Σ_0_ and some constant *δ*_0_. Equivalently, this spatio-temporal scale-space representation at scale *τ* can be obtained by convolution with *temporal Gaussian kernels* of the form

$$\begin{array}{c} g\left(t;\;\tau \right)=\frac{1}{\sqrt{2\pi \Sigma_{\tau }}}\;e^{-{\left(t-\delta_{\tau }\right)}^{2}/2\tau } \end{array}$$ (26)

with Σ_*τ*_ = *τ*Σ_0_ and *δ*_*τ*_ = *τ* *δ*_0_. Since the parameter Σ_0_ only corresponds to an unessential rescaling of the temporal scale parameter *τ*, we will set Σ_0_ = 1.

To verify that the solutions of the diffusion equation obey non-enhancement of local extrema is straightforward. For a one-dimensional signal the first-order derivative is zero, whereas the value of the second derivative will be positive at local minima and negative at local maxima. Hence, for Gaussian smoothing as governed by the diffusion Equation (25), the derivative with respect to scale is guaranteed to be positive at local minima and negative at local maxima (sufficiency). A less immediate result is that non-enhancement of local extrema also implies that the evolution over scale must be governed by the diffusion equation (necessity) and is proved in (Lindeberg [14]).

**Fig 2:**
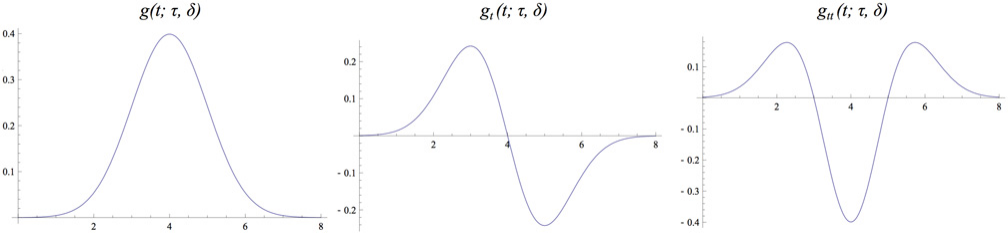
*The time-shifted Gaussian kernel* $g(t;\tau ,\delta )=1/\sqrt{2\pi \tau }\exp(-{\left(t-\delta \right)}^{2}/2\tau )$ for *τ* = 1 and *δ* = 4 with its first- and second-order temporal derivatives.

Graphs of these Gaussian kernels are shown in fig. 2. Notably, these kernels are not strictly time causal. To arbitrary degree of accuracy, however, they can be approximated by truncated time-causal kernels, provided that the time delay *δ* is chosen sufficiently long in relation to the temporal scale *τ*. Hence, the choice of *δ* leads to a trade-off between the computational accuracy of the implementation and the temporal response properties as delimited by a non-zero time delay. This problem, however, arises only for real-time analysis. For off-line computations, the time delay may be set to zero, corresponding to kernels that are mirror symmetric *T*(−*t*; *s*) = *T*(*t*; *s*) through the origin. Thus, the truncated and time-shifted Gaussian kernels can serve as a simplest possible model for a temporal scale-space representation, provided that the requirements of temporal causality and temporal recursivity can be relaxed.

#### Derived receptive fields in terms of temporal derivatives

In addition to the zero-order smoothing kernel *T*, we have in fig. 2 also shown its first- and second-order temporal derivatives *T*_*t*_ and *T*_*tt*_. Such derivatives of scale-space kernels do also obey desirable structural properties in terms of linearity, shift invariance and nice properties over scale in terms of non-enhancement of local extrema, with the semi-group property replaced by a cascade property over scale

$$\begin{array}{c} \left(\partial_{t^{\alpha }}L\right)\left(\cdot ;\;\tau_{2}\right)=T\left(\cdot ;\;\tau_{2}-\tau_{1}\right)*\left(\partial_{t^{\alpha }}L\right)\left(\cdot ;\;\tau_{1}\right) \end{array}$$ (27)

and with the limit case when the temporal scale goes to zero (14) replaced by

$$\begin{array}{c} \lim_{\tau ↓0}\left(\partial_{t^{\alpha }}L\right)\left(\cdot ;\;\tau \right)=\lim_{\tau ↓0}\partial_{t^{\alpha }}\left({𝓣}_{\tau }f\right)=\partial_{t^{\alpha }}f \end{array}$$ (28)

provided that the corresponding derivative of *f* exists. Regarding temporal receptive fields that are expressed in terms of derivatives of scale-space kernels, the normalization condition (9) is replaced by the integral of the receptive field being zero

$$\begin{array}{c} ∥\left(\partial_{t^{\alpha }}T\right)\left(\cdot ;\;\tau \right){∥}_{1}=\int_{t\in \mathbb{R}}\left(\partial_{t^{\alpha }}T\right)\left(t;\;\tau \right)\;dt=0. \end{array}$$ (29)

In all other major respects, such receptive fields satisfy essential scale-space properties in terms of non-creation of new structures with increasing scale in the sense that local extrema in the receptive field response are not enhanced from a fine to a coarser scale or that the number of local extrema or zero-crossings in the signal is guaranteed to not increase from any fine to any coarser scale.

Additionally, receptive fields that are expressed in terms of temporal derivatives are invariant under additive transformations of the signal

$$\begin{array}{c} f(t)\mapsto f(t)+C \end{array}$$ (30)

and thereby provide a mechanism for capturing local variations in the signal under variabilities of its baseline.

### Time-causal temporal scale-space

When constructing a system for real-time processing of sensory data, a fundamental constraint on the temporal smoothing kernels is that they have to be *time-causal*. The ad hoc solution of using a truncated symmetric filter of finite temporal extent in combination with a temporal delay is not appropriate in a time-critical context. Because of computational and memory efficiency, the computations should furthermore be based on a compact temporal buffer that contains sufficient information for representing sensory information at multiple temporal scales and computing features therefrom. Corresponding requirements are necessary in computational modelling of biological perception.

#### Time-causal scale-space kernels for pure temporal domain

Given the requirement on temporal scale-space kernels by non-creation of local extrema over a pure temporal domain, *truncated exponential kernels*

$$\begin{array}{c} h_{exp}\left(t;\;\mu_{k}\right)=\left\{\begin{array}{cc} \frac{1}{\mu_{k}}e^{-t/\mu_{k}} & t\geq 0\\ 0 & t<0 \end{array}\right. \end{array}$$ (31)

can be shown to constitute the only class of time-causal scale-space kernels over a continuous domain (Lindeberg [42]; Lindeberg and Fagerström [44]). The Laplace transform of such a kernel is given by

$$\begin{array}{c} H_{exp}\left(q;\;\mu_{k}\right)=\int_{t=-\infty }^{\infty }h_{exp}\left(t;\;\mu_{k}\right)\;e^{-qt}\;dt=\frac{1}{1+\mu_{k}q} \end{array}$$ (32)

and coupling *K* such kernels in cascade leads to a composed filter

$$\begin{array}{c} h_{composed}\left(t;\;\mu \right)={*}_{k=1}^{K}h_{exp}\left(t;\;\mu_{k}\right) \end{array}$$ (33)

having a Laplace transform of the form

$$\begin{array}{cc} H_{composed}\left(q;\;\mu \right) & =\int_{t=-\infty }^{\infty }\left({*}_{k=1}^{K}h_{exp}\left(t;\;\mu_{k}\right)\right)\;e^{-qt}\;dt=\prod_{k=1}^{K}\frac{1}{1+\mu_{k}q} \end{array}$$ (34)

The composed filter has temporal mean and variance

$$\begin{array}{cc} m_{K} & =M\left(h_{composed}\left(\cdot ;\;\mu \right)\right)=\sum_{k=1}^{K}\mu_{k} \end{array}$$ (35)

$$\begin{array}{cc} \tau_{K} & =V\left(h_{composed}\left(\cdot ;\;\mu \right)\right)=\sum_{k=1}^{K}\mu_{k}^{2} \end{array}$$ (36)

**Fig 3:**
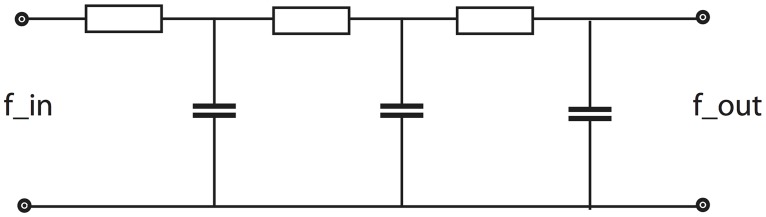
Electric wiring diagram consisting of a set of resistors and capacitors that emulate a series of first-order integrators coupled in cascade. Here, we regard the time-varying voltage *f*_*in*_ as representing the time varying input signal and the resulting output voltage *f*_*out*_ as representing the time varying output signal at a coarser temporal scale. According to the theory of temporal scale-space kernels for one-dimensional signals (Lindeberg [42]; Lindeberg and Fagerström [44]), the corresponding equivalent truncated exponential kernels are the only primitive temporal smoothing kernels that guarantee both temporal causality and non-creation of local extrema (alternatively zero-crossings) with increasing temporal scale. Such first-order temporal integration can be used as a straightforward computational model for temporal processing in biological neurons; see also (Koch [46], chapters 11–12) regarding physical modelling of the information transfer in dendrites of neurons.

In terms of physical models, repeated convolution with such kernels corresponds to coupling a series of *first-order integrators* with time constants *μ*_*k*_ in cascade:

$$\begin{array}{c} \partial_{t}L\left(t;\;\tau_{k}\right)=\frac{1}{\mu_{k}}\left(L\left(t;\;\tau_{k-1}\right)-L\left(t;\;\tau_{k}\right)\right) \end{array}$$ (37)

with *L*(*t*; 0) = *f*(*t*). These temporal smoothing kernels satisfy scale-space properties in the sense that the number of local extrema or the number of zero-crossings in the temporal signal are guaranteed to not increase with the temporal scale. In this respect, these kernels have a desirable and well-founded smoothing property that can be used for defining multi-scale observations over time. A limitation of this type of temporal scale-space representation, however, is that the *scale levels are required to be discrete* and that the scale-space representation does hence not admit a continuous scale parameter. Computationally, however, the scale-space representation based on truncated exponential kernels can be highly efficient and admits for direct implementation in terms of hardware (or wetware) that emulates first-order integration over time (see fig. 3 for an illustration of a corresponding electric wiring diagram).

When implementing this temporal scale-space concept, a set of intermediate scale levels has to be distributed between some minimum and maximum scale levels *τ*_*min*_ and *τ*_*max*_. Assuming that a total number of *K* scale levels is to be used, it is natural to distribute the temporal scale levels according to a geometric series, corresponding to a uniform distribution in units of *effective temporal scale* *τ*_*eff*_ = log*τ* (Lindeberg [47]). Using such a logarithmic distribution of the temporal scale levels, the different levels in the temporal scale-space representation at increasing temporal scales will serve as a logarithmic memory of the past, with qualitative similarity to the mapping of the past onto a logarithmic time axis in the scale-time model by Koenderink [48]. If we have the freedom of choosing *τ*_*min*_ freely, a natural way of parameterizing these temporal scale levels is by using a distribution parameter *c* > 1 such that

$$\begin{array}{c} \tau_{k}=c^{2(k-K)}\tau_{max}\;\;\left(1\leq k\leq K\right) \end{array}$$ (38)

which by Equation (36) implies that time constants of the individual first-order integrators will be given by

$$\begin{array}{cc} \mu_{1} & =c^{1-K}\sqrt{\tau_{max}} \end{array}$$ (39)

$$\begin{array}{cc} \mu_{k} & =\sqrt{\tau_{k}-\tau_{k-1}}=c^{k-K-1}\sqrt{c^{2}-1}\sqrt{\tau_{max}}\;\;\left(2\leq k\leq K\right) \end{array}$$ (40)

If the temporal signal is on the other hand given at some minimum temporal scale level *τ*_*min*_, we can instead determine *c* in (38) such that *τ*_1_ = *τ*_*min*_

$$\begin{array}{c} c={\left(\frac{\tau_{max}}{\tau_{min}}\right)}^{\frac{1}{2(K-1)}} \end{array}$$ (41)

and add *K* − 1 temporal scale levels with *μ*_*k*_ according to (40). Alternatively, if one chooses a uniform distribution of the intermediate temporal scale levels

$$\begin{array}{c} \tau_{k}=\frac{k}{K}\;\tau_{max} \end{array}$$ (42)

implying

$$\begin{array}{c} \mu_{k}=\mu =\sqrt{\frac{\tau_{max}}{K}}, \end{array}$$ (43)

then it becomes straightforward to compute the explicit expression for the composed kernel

$$\begin{array}{c} h_{composed}\left(t;\;\mu ,k\right)={ℒ}^{-1}\left(\frac{1}{{\left(1+\mu q\right)}^{k}}\right)=\frac{t^{k-1}\;e^{-t/\mu }}{\mu^{k}\;\Gamma \left(k\right)} \end{array}$$ (44)

having temporal mean value *m*_*k*_ = *k* *μ* and variance *τ* = *k* *μ* ^2^. In contrast to the primitive truncated exponentials, which are discontinuous at the origin, these kernels are continuous of order *k* − 1, allowing for differentiation up to order *k* − 1. The corresponding expressions for the first- and second-order derivatives are:

$$\begin{array}{ccc} h_{composed,t}\left(t;\;\mu ,k\right) & = & \mu^{-k-1}t^{k-2}\frac{\left((k-1)\mu -t\right)}{\Gamma (k)}e^{-t/\mu }\\ & = & -\frac{\left(t-(k-1)\mu \right)}{\mu t}\times h_{composed,t}\left(t;\;\mu ,k\right), \end{array}$$ (45)

$$\begin{array}{ccc} h_{composed,tt}\left(t;\;\mu ,k\right) & = & \mu^{-k-2}t^{k-3}\frac{\left(\left(k^{2}-3k+2\right)\mu^{2}-2\left(k-1\right)t\mu +t^{2}\right)}{\Gamma (k)}e^{-t/\mu }\\ & = & \frac{\left(\left(k^{2}-3k+2\right)\mu^{2}-2\left(k-1\right)t\mu +t^{2}\right)}{\mu^{2}t^{2}}\times h_{composed,t}\left(t;\;\mu ,k\right). \end{array}$$ (46)

**Fig 4:**
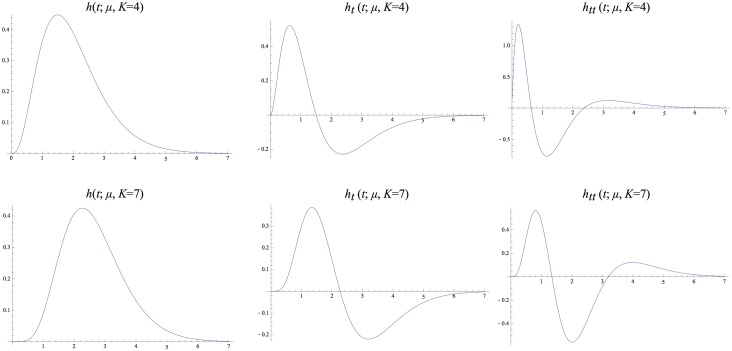
*Equivalent kernels* $h_{composed}(t;\;\mu )={*}_{k=1}^{K}h_{exp}(t;\;\mu )$ *with temporal variance τ* = 1 *corresponding to the composition of K truncated exponential kernels with equal time constants μ* and their first- and second-order derivatives. (top row) *k* = 4 and $\mu =\sqrt{1/4}$. (bottom row) *k* = 7 and $\mu =\sqrt{1/7}$.

**Fig 5:**
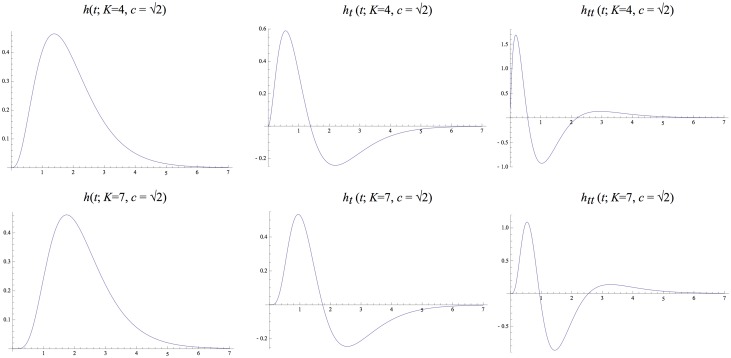
*Equivalent kernels* $h_{composed}(t;\;\mu )={*}_{k=1}^{K}h_{exp}(t;\;\mu_{k})$ *with temporal variance τ* = 1 *corresponding to the composition of K* = 4 *or K* = 7 *truncated exponential kernels with different time constants defined from a self-similar distribution of the temporal scale levels* according to Equations (38), (39) and (40) and corresponding to a uniform distribution in terms of effective temporal scale *τ*_*eff*_ = log *τ* for $c=\sqrt{2}$ and with their first- and second-order derivatives.

**Fig 6:**
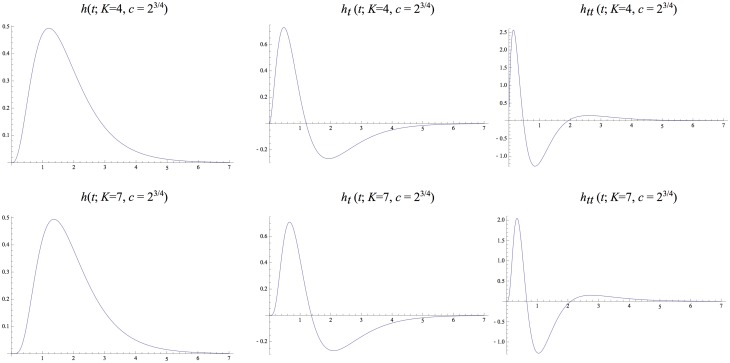
*Equivalent kernels* $h_{composed}(t;\;\mu )={*}_{k=1}^{K}h_{exp}(t;\;\mu_{k})$ *with temporal variance τ* = 1 *corresponding to the composition of K* = 4 *or K* = 7 *truncated exponential kernels with different time constants defined from a self-similar distribution of the temporal scale levels* according to Equations (38), (39) and (40) and corresponding to a uniform distribution in terms of effective temporal scale *τ*_*eff*_ = log *τ* for *c* = 2^3/4^ and with their first- and second-order derivatives.

Fig. 4 shows graphs of these kernels for two combinations of *μ* and *K* that correspond to the same value of the composed variance *τ* = *K* *μ* ^2^. Notably, these kernels are highly asymmetric for small values of *K*, whereas they become gradually more symmetric as *K* increases. Figs. 5–6 show corresponding compositions of truncated exponential kernels for self-similar distributions of the intermediate time constants according to Equations (38), (39) and (40) for $c=\sqrt{2}$ and *c* = 2^3/4^. Comparing fig. 4 and figs. 5–6, the use of a self-similar distribution of the time constants (in figs. 5–6) allows for smoother behaviour near the origin with increasing *K* while not increasing the temporal delay as much as for the kernels corresponding to a uniform distribution of the intermediate temporal scale levels (in fig. 4).

#### Time-recursive computation of temporal derivatives

Temporal scale-space derivatives of order *r* can be defined from this scale-space model according to

$$\begin{array}{c} L_{t^{r}}\left(\cdot ;\;\tau_{K}\right)=\partial_{t^{r}}L\left(\cdot ;\;\tau_{K}\right)=(\partial_{t^{r}}\left({*}_{k=1}^{K}h_{exp}\left(t;\;\mu_{k}\right)\right)*f \end{array}$$ (47)

where the Laplace transform of the composed (equivalent) derivative kernel is

$$\begin{array}{c} H_{composed}^{\left(r\right)}\left(q;\;\tau_{K}\right)=q^{r}\prod_{k=1}^{K}\frac{1}{1+\mu_{k}q} \end{array}$$ (48)

For this kernel to have a net integration effect (to enable well-posed derivative operators), the total order of differentiation must not exceed the total order of integration. Thereby, *r* < *k* is a necessary requirement. The composed transfer function must have finite *L*_2_-norm.

A very useful observation that can be made concerning derivative computations is that temporal derivatives can equivalently be computed from differences between different temporal channels. Let us first assume that all time constants *μ*_*i*_ are different in (48). Then, a partial fraction division gives

$$\begin{array}{c} H_{composed}^{\left(r\right)}\left(q;\;\tau_{k}\right)=\sum_{i=1}^{k}A_{i}\;H_{prim}\left(q;\;\mu_{i}\right) \end{array}$$ (49)

where

$$\begin{array}{c} A_{i}=\frac{{\left(-1\right)}^{r}}{\mu_{i}^{r}}\prod_{j=1,j\neq i}^{k}\frac{1}{\left(1-\mu_{j}/\mu_{i}\right)}\;\;\left(1\leq i\leq k\right) \end{array}$$ (50)

showing that *each temporal derivative can be computed as a linear combination of the representations at the different time-scales*.

More realistically, the channels that we can regard as available at a certain temporal scale with index *k* will not be the results of direct filtering with different time constants *μ*_*i*_. Rather, we would like to use the intermediate outputs from the cascade coupled recursive filters *H*_*composed*_(*q*; *τ*_*i*_) for *k* − *r* ≤ *i* ≤ *k*. Decomposition of $H_{composed}^{\left(r\right)}$ into a sum of *r* such transfer functions

$$\begin{array}{c} H_{composed}^{\left(r\right)}\left(q;\;\tau_{k}\right)=\sum_{i=k-r}^{k}B_{i}\;H_{composed}\left(q;\;\tau_{i}\right) \end{array}$$ (51)

shows that the weights *B*_*i*_ are given as the solution of a triangular system of equations provided that the necessary condition *r* < *k* is satisfied

$$\begin{array}{c} \frac{{\left(-1\right)}^{r}}{\mu_{i}^{r}}\prod_{j=i+1}^{k}\frac{1}{\left(1-\mu_{j}/\mu_{i}\right)}=B_{i}+\sum_{\nu =i+1}^{k}B_{\nu }\prod_{j=i+1}^{\nu }\frac{1}{\left(1-\mu_{j}/\mu_{i}\right)}\;\;\left(k-r\leq i\leq k\right). \end{array}$$ (52)

It can be shown that the Laplace transforms of the equivalent derivative computation kernels satisfy the following recurrence relation (Lindeberg and Fagerström [44])

$$\begin{array}{c} H_{composed}^{\left(r\right)}\left(q;\;\tau_{k}\right)=\frac{1}{\mu_{k}}\left(H_{composed}^{\left(r-1\right)}\left(q;\;\tau_{k-1}\right)-H_{composed}^{\left(r-1\right)}\left(q;\;\tau_{k}\right)\right) \end{array}$$ (53)

implying that higher-order temporal derivatives can be computed from small-support finite differences of lower-order derivatives, where the temporal scale-space representations at different temporal scales serve as a sufficient temporal buffer of what has occurred in the past. Derivative computations will therefore be highly efficient. Both the temporal smoothing operation and the computation of temporal derivatives are time-recursive.

## Multi-scale spectrograms for auditory signals

The above treatment concerning is general and can be used for modelling desirable properties of temporal receptive fields for a variety of time-dependent sensory signals. For auditory signals, an additional structural requirement arises from the fact that the auditory information is transferred in terms of sound waves that travel from the transmitter to the receiver and the auditory information can be encoded in terms of oscillation frequencies of the air pressure that generates the sensory signal. For this reason and from our knowledge that the variations due to the geometry and other properties of the cochlea leads to physical resonances whose effect can be modelled as a physical Fourier transform, *spectrograms* are a common tool for analyzing auditory information.

Note that our primary aim is not to specifically model, for example, the measured response of the nerves coming from the cochlea as typically done in previous auditory models (Patterson et al. [36]). Instead we are following the scale-space theory using the principle of invariance as outlined in the sections “Structural requirements on temporal receptive fields” and “Scale-space concepts for purely temporal domains”.

Based on the two models for temporal receptive fields (non-causal in the section “Non-causal Gaussian temporal scale-space” and time-causal in the section “Time-causal temporal scale-space”), we can use the temporal smoothing functions in these two temporal scale-space models as scale-dependent window functions for defining two types of complex-valued *multi-scale spectrograms* according to

$$\begin{array}{ccc} S_{g}\left(t,\omega ;\;\tau \right) & = & \int_{t^{'}=-\infty }^{\infty }g\left(t-t^{'};\;\tau \right)\;f\left(t^{'}\right)\;e^{-i\omega t^{'}}\;dt^{'} \end{array}$$ (54)

$$\begin{array}{ccc} S_{h}\left(t,\omega ;\;\mu \right) & = & \int_{t^{'}=-\infty }^{\infty }h_{composed}\left(t-t^{'};\;\mu \right)\;f\left(t^{'}\right)\;e^{-i\omega t^{'}}\;dt^{'} \end{array}$$ (55)

where

- *g*(*t*; *τ*) is a temporal Gaussian kernel of the form (26),
- *h*_*composed*_(*t*; *μ*) with *μ* = (*μ*_1_, …, *μ*_*K*_) is the equivalent convolution kernel corresponding to a cascade of truncated exponential filters of the form (33).

This implies that the convolution kernels in the temporal scale-spaces for a general time-varying signal are used as scale-dependent window functions for defining windowed Fourier transforms of different temporal extent.

For a given value of *τ*, the spectrogram becomes a 2-D function. With the definition extended to all values of *τ*, the spectrogram based on Gaussian window functions instead becomes a 3-D volume over all temporal extents of the window function or alternatively a set of discrete 2-D slices for the window functions based on truncated exponential functions coupled in cascade for vectors *μ* = (*μ*_1_, …, *μ*_*K*_) of different length *K*.

Note that *a priori* there may be no principled reason for preferring a particular duration of the temporal window function for the windowed Fourier transform over some other temporal duration. Specifically, different temporal durations may be appropriate for different auditory tasks, such as a preference for a short temporal duration for onset detection and a preference for a longer temporal duration to separate sounds with nearby frequencies. Thereby, the scale-space approach allows for the definition of windowed Fourier transforms for all temporal extents in such a way that any windowed Fourier transform at a coarse temporal scale can be related to a windowed Fourier transform at any finer scale using the *cascade property*

$$\begin{array}{ccc} S(\cdot ,\omega ;\;\tau_{2}) & = & w\left(\cdot ;\;\tau_{2}-\tau_{1}\right)*S\left(\cdot ,\omega ;\;\tau_{1}\right), \end{array}$$ (56)

$$\begin{array}{ccc} S(\cdot ,\omega ;\;\tau_{n}) & = & \left(\Delta w\right)\left(\cdot ;\;m\mapsto n\right)*S\left(\cdot ,\omega ;\;\tau_{m}\right). \end{array}$$ (57)

derived from the semi-group structure (15) or the Markov property (18) of the underlying scale-space kernels. Combined with the additional scale-space properties of non-creation of new structures with increasing scale, this guarantees well-founded theoretical properties between corresponding windowed Fourier transforms at different temporal scales.

In most other work on auditory signal processing, there is often an implicit assumption that one chooses a scale for computing the auditory features that seems to work and on which later stage analysis is then based. By the presented formulation of multi-scale spectrograms, we aim at making the consequences of such assumptions explicit, and emphasizing the possibility of computing auditory features at multiple temporal scales as an integrated part of the analysis. Compared to the more traditional approach of computing spectrograms from local fast Fourier transforms combined with local windowing operations, this formulation of multi-scale spectrograms also avoids the concatenation of such windowing operations altogether and thereby the artifacts caused by these.

The scale-space approach for defining multi-scale auditory spectrograms implies that instead of computing a scale-space representation of the original auditory signal, the auditory signal is first projected onto the two orthogonal dimensions cos*ωt* and *i*sin*ωt* of a complex sine wave *e* ^−*iωt*^

$$\begin{array}{c} f_{cos}\left(t,\omega \right)=f\left(t\right)\;\cos\omega t\;\;f_{sin}\left(t,\omega \right)=f\left(t\right)\;\sin\omega t \end{array}$$ (58)

for which temporal scale-space representations are then defined, implying that the multi-scale spectrogram can be seen as a complex-valued scale-space transform.

### 

#### Invariance and covariance properties

Concerning the symmetry requirements of a general temporal sensory front-end described in the section “Structural requirements on temporal receptive fields”, the linearity of the scale-space operations is transferred to a linearity in the complex multi-scale spectrograms (54)–(55). This implies that multiple sources of sound will be combined in an additive manner in terms of their complex-valued responses and that sound sources of different strength (sound pressure) will be handled in a similar manner up to a multiplication of the strength of the signal.

Regarding temporal shift invariance, the magnitude maps ∣*S*_*g*_∣ and ∣*S*_*h*_∣ are invariant under a shift of the temporal axis, whereas the phase of the truly complex spectrograms *S*_*g*_ and *S*_*h*_ will be transformed in a predictable manner between similar sound signals at different time moments or from different distances to the observer.

Under a local rescaling of the temporal axis

$$\begin{array}{c} t\mapsto \alpha \;t \end{array}$$ (59)

the temporal receptive fields from the Gaussian scale-space model are fully scale covariant and the corresponding complex-valued multi-scale spectrograms are transformed according to

$$\begin{array}{cc} S(t,\omega ;\;\tau ) & \mapsto S(\alpha \;t,\frac{\omega }{\alpha };\;\alpha^{2}\tau ). \end{array}$$ (60)

If we let the window scale $\sigma =\sqrt{\tau }$ for any angular frequency *ω* be proportional to the wavelength *λ* = 2*π*/*ω* corresponding to that frequency, then the corresponding spectrograms within the same 2-D slice of the extended 3-D multi-scale spectrogram can therefore be matched by a corresponding frequency shift

$$\begin{array}{c} \omega \mapsto \frac{\omega }{\alpha }. \end{array}$$ (61)

If the temporal window functions on the other hand do not have the temporal extent proportional to the wavelength, then temporal covariance does not hold within the same 2-D slice but still holds within the 3-D multi-scale spectrogram based on Gaussian window functions because of their self-similarity over scale, whereas the corresponding scaling relations can only be approximate for the truncated exponential functions coupled in cascade, because of the temporal scale levels being restricted to a discrete set of values.

Again there may not be any principled reason for preferring a particular temporal scale over another. The multi-scale nature of these spectrograms makes this aspect explicit and opens up for using different temporal scales for different auditory tasks, where different temporal scales may have complementary advantages.

##### Relations to Gabor functions

By rewriting the expression (54) for the complex-valued spectrogram based on the Gaussian temporal scale-space concept as

$$\begin{array}{c} S_{g}\left(\omega ,t;\;\tau \right)=e^{-i\omega t}\int_{t^{'}=-\infty }^{\infty }g\left(t-t^{'};\;\tau \right)\;e^{i\omega (t-t^{'})}\;f\left(t^{'}\right)\;dt^{'}, \end{array}$$ (62)

it can be seen that up to a phase shift, this multi-scale spectrogram can equivalently be interpreted as the convolution of the original auditory signal *f* by *Gabor functions* [28] of the form

$$\begin{array}{c} G\left(t,\omega ;\;\tau \right)=g\left(t;\;\tau \right)\;e^{i\omega t}. \end{array}$$ (63)

Such Gabor functions have been previously used for analyzing auditory signals by several authors, including (Wolfe et al. [29]; Kleinschmidt et al. [49, 50]; Lobo and Loizou [30]; Qiu et al. [31]; van de Boogart and Lienhart [51]; Ezzat et al. [52]; Domont et al. [53]; He et al. [54]; Heckmann et al. [55]; Wu et al. [32]; Schädler et al. [56]; Sameh and Lachiri [57]). Our theory provides a new way of deriving this representation with special emphasis on the multi-scale nature of the Gaussian window functions and their resulting cascade properties between spectrograms at different temporal scales.

##### Relations to Gammatone filters

In the special case when the time constants of all the *K* truncated exponential filters that are coupled in cascade are all equal *μ*_*k*_ = *μ*, it follows from combination of Equations (55) and (44) that the multi-scale spectrogram is given by

$$\begin{array}{c} S_{h}\left(t,\omega ;\;\mu ,K\right)=e^{-i\omega t}\int_{t^{'}=-\infty }^{\infty }\frac{{\left(t-t^{'}\right)}^{K-1}\;e^{-(t-t^{'})/\mu }}{\mu^{K}\;\Gamma \left(K\right)}\;e^{i\omega (t-t^{'})}\;f\left(t^{'}\right)\;dt^{'} \end{array}$$ (64)

and does up to a phase shift correspond to convolution of the input signal *f* by filters of the form

$$\begin{array}{ccc} h_{cos}\left(t,\omega ;\;\mu ,K\right) & = & \frac{t^{K-1}\;e^{-t/\mu }}{\mu^{K}\;\Gamma \left(K\right)}\cos\omega t, \end{array}$$ (65)

$$\begin{array}{ccc} h_{sin}\left(t,\omega ;\;\mu ,K\right) & = & \frac{t^{K-1}\;e^{-t/\mu }}{\mu^{K}\;\Gamma \left(K\right)}\sin\omega t. \end{array}$$ (66)

For comparison, the *Gammatone filter* with parameters *a* and *b* and frequency *ϕ* is defined according to

$$\begin{array}{c} \gamma \left(t\right)=a\;t^{n-1}e^{-2\pi bt}\cos\left(2\pi \phi \;t+\alpha \right). \end{array}$$ (67)

By identification of the parameters

$$\begin{array}{c} a=\frac{1}{\mu^{K}\;\Gamma \left(K\right)}\;\;b=\frac{1}{2\pi \mu } \end{array}$$ (68)

and using *ω* = 2*π* *ϕ* it follows that we can derive the Gammatone filter as a special case of applying a time-causal scale-space representation with discrete scale levels to the projections *f*_*cos*_(*t*, *ω*) and *f*_*sin*_(*t*, *ω*) of an auditory signal *f*(*t*) onto a complex sine wave *e* ^−*iωt*^.

Gammatone filter banks are also commonly used in audio processing (Johannesma [33]; Patterson et al. [34, 36]; Hewitt and Meddis [35]; Irino and Patterson [58]; Ambikairajah [59]; Hohmann [60]; van Immerseel and Peeters [61]; Schlute et al. [62]; Ngamkham et al. [63]). The present treatment provides a new way of deriving them in a principled and conceptually similar way as the Gabor filters can be derived, with the differences that the temporal filtering operations are required to be truly time-causal and that only a discrete set of temporal scale levels is to be used.

##### Generalized Gammatone filters

In addition, by allowing for different time constants in the primitive truncated exponential filters, this scale-space concept leads to *generalized Gammatone filters*

$$\begin{array}{ccc} h_{cos}\left(t,\omega ;\;\mu \right) & = & h_{composed}\left(t;\;\mu \right)\;\cos\omega t \end{array}$$ (69)

$$\begin{array}{ccc} h_{sin}\left(t,\omega ;\;\mu \right) & = & h_{composed}\left(t;\;\mu \right)\;\sin\omega t \end{array}$$ (70)

with *h*_*composed*_ according to (34). By comparing graphs of the two classes of auditory receptive fields based on time-causal window functions (Lindeberg and Friberg [64], fig. 6), it can be seen that the frequency selective filters based on truncated exponential filters having a logarithmic distribution of the intermediate temporal scale levels allow for a faster temporal response compared to the corresponding filters based on truncated exponential filters with equal time constants. Thereby, these generalized Gammatone filters allow for additional degrees of freedom to obtain different trade-offs between the frequency selectivity and the temporal delay of time-causal window functions by varying the number of levels *K* and the distribution parameter *c*—see the appendices “Frequency selectivity of the spectrograms” and “Temporal dynamics of the time-causal kernels” for an in-depth analysis of the frequency selectivity and the temporal delay of such kernels.

**Fig 7:**
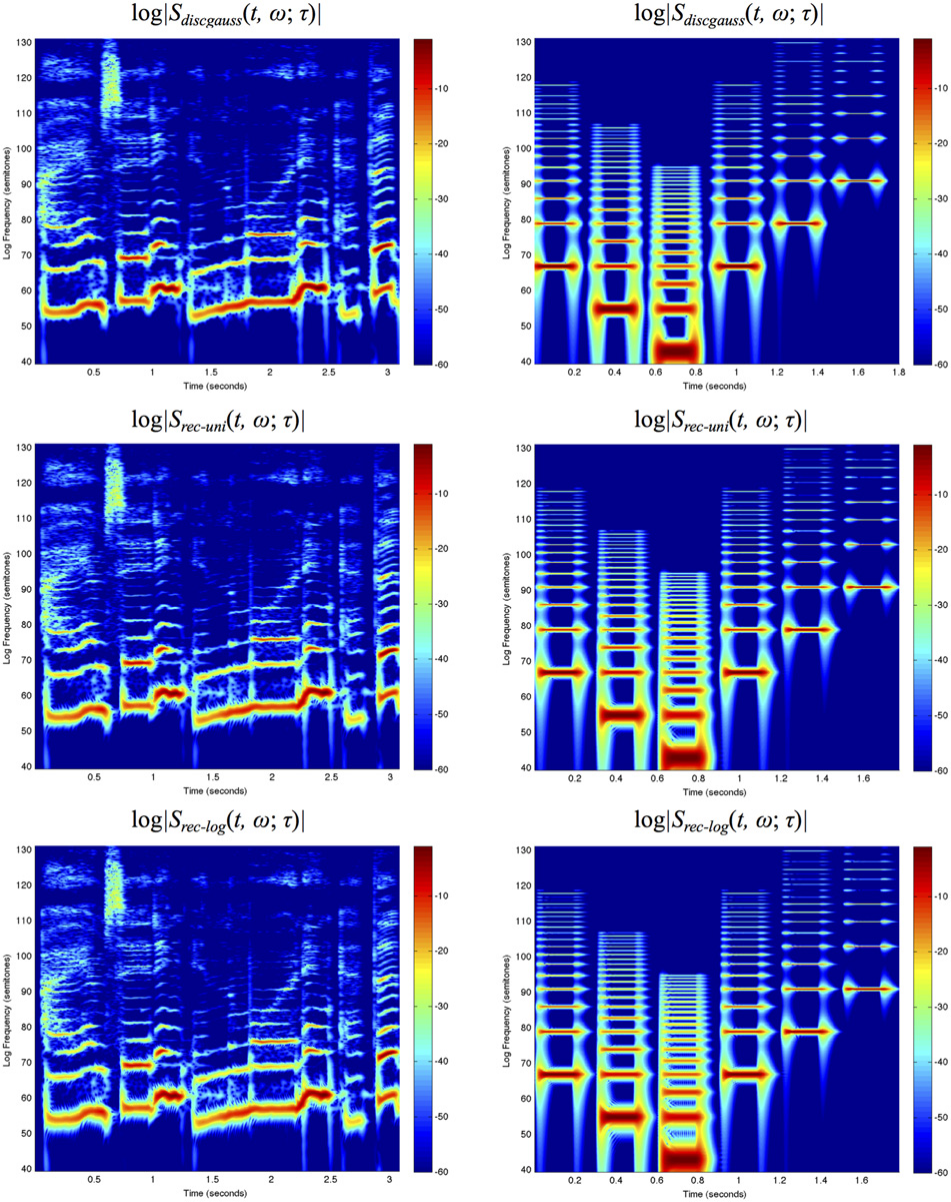
*Spectrograms with a fixed temporal window scale* $\sigma_{t}=\sqrt{\tau }=20\;\text{ms}$ computed using different temporal scale-space concepts for (left column) the first 3 seconds of “Tom’s diner” by Suzanne Vega with the lyrics “I am sitting in the morning at the …” and (right column) a synthetic signal containing harmonic spectra with different fundamental frequencies over a logarithmic frequency scale from 80 Hz to 16 kHz using 48 frequency levels per octave: (top row) the discrete analogue of the Gaussian kernel, (middle row) a cascade of seven time-causal recursive filters having a uniform distribution of the temporal scale levels and (bottom row) a cascade of seven time-causal recursive filters having a logarithmic distribution of the temporal scale levels with $c=\sqrt{2}$. The vertical axis shows the logarithmic frequency expressed in semitones with 69 corresponding to the tone A4 (440 Hz). Notice that while the different types of spectrograms largely capture similar types of spectro-temporal structures, there is a significant difference in temporal delay and temporal response characteristics between the non-causal and the time-causal spectrograms.

**Fig 8:**
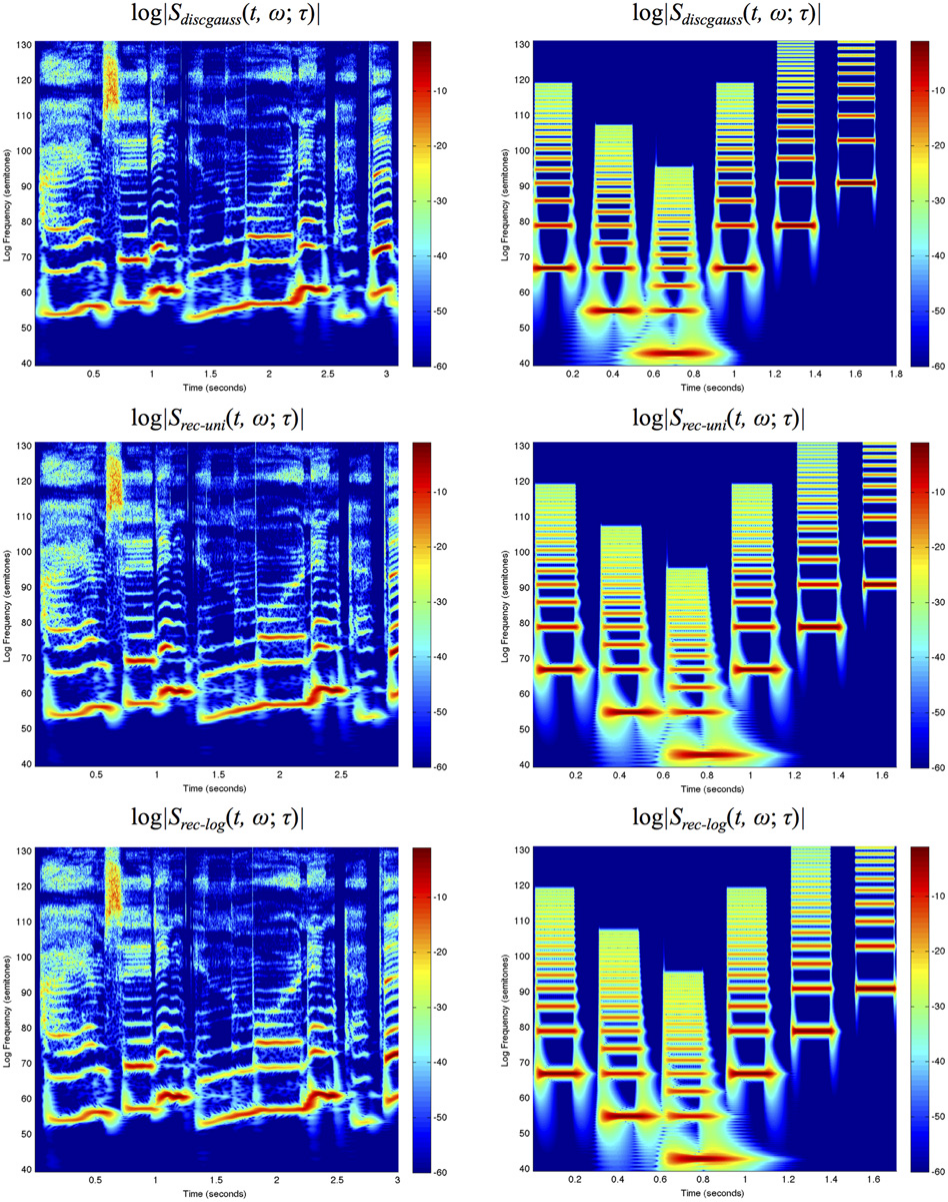
*Spectrograms with the temporal window scale proportional to the wavelength according to* Equation (73) for *n* = 8 and a soft lower threshold $\sigma_{0}=\sqrt{\tau_{0}}=1\;\text{ms}$ computed using different temporal scale-space concepts for (left column) the first 3 seconds of “Tom’s diner” by Suzanne Vega with the lyrics “I am sitting in the morning at the …” and (right column) a synthetic signal containing harmonic spectra with different ground tones over a logarithmic frequency range from 80 Hz to 16 kHz using 48 frequency levels per octave: (top row) the discrete analogue of the Gaussian kernel, (middle row) a cascade of seven time-causal recursive filters having a uniform distribution of the temporal scale levels and (bottom row) a cascade of seven time-causal recursive filters having a logarithmic distribution of the temporal scale levels with $c=\sqrt{2}$. The vertical axis shows the logarithmic frequency expressed in semitones with 69 corresponding to the tone A4 (440 Hz). The spectrograms computed with time-causal kernels have been delay compensated by a temporal delay defined from the position of the first inflection point of the temporal window function. Notice that while the different types of spectrograms to some extent capture qualitatively similar types of spectro-temporal structures, there is a significant difference in temporal delay and temporal response characteristics between the non-causal and the time-causal spectrograms. Compared to the spectrograms in fig. 7 that are computed with a fixed temporal scale implying that the spectral bands become more narrow at higher frequencies, the use of a temporal scale proportional to the wavelength specifically implies that the widths of the spectral bands are here much more uniform over frequencies (see the section “Frequency selectivity of the spectrogram” for a theoretical analysis).

Figs. 7–8 show spectrograms computed in this way for two sound signals using the three different types of temporal window functions and using fixed *vs*. frequency-dependent window scales.

##### Frequency-dependent window scale

To guarantee basic covariance properties of the spectrogram under a frequency shift

$$\begin{array}{c} \omega \mapsto \alpha \;\omega \end{array}$$ (71)

it is as earlier mentioned natural to let the temporal window scale vary with the frequency *ω* in such a a way that the temporal window scale in units of $\sigma =\sqrt{\tau }$ is *proportional to the wavelength* *λ* = 2*π*/*ω*

$$\begin{array}{c} \tau ={\left(\frac{2\pi \;n}{\omega }\right)}^{2} \end{array}$$ (72)

where *n* is a parameter. By such frequency dependency of the temporal window scale, the spectral selectivity in the spectrogram (the width of a spectral band) will be independent of the frequency *ω* (see the section “Frequency selectivity of the spectrograms”). This is a prerequisite for the desirable property that a shift by one octave of a musical piece should imply that the corresponding spectrogram should appear similar while shifted by one octave, if the frequency axis of the spectrogram is parameterized on a logarithmic scale.

To prevent the temporal window scale from being too short for high frequency sounds, we have additionally chosen to add a *soft lower bound* such that the temporal extent is instead chosen according to

$$\begin{array}{c} \tau =\tau_{0}+{\left(\frac{2\pi \;n}{\omega }\right)}^{2} \end{array}$$ (73)

where $\tau_{0}=\sigma_{0}^{2}$ denotes a lower bound on the temporal window scale. Thereby, frequency covariance of a 2-D spectrogram will only be approximate, while being a good approximation if *τ* ≫ *τ*_0_. If we quantify *τ* ≫ *τ*_0_ as *τ* = *β* ^2^ *τ*_0_, then the soft lower bound corresponds to

$$\begin{array}{c} \omega =\frac{2\pi n}{\sqrt{\beta^{2}-1}\;\sigma_{0}} \end{array}$$ (74)

which with *σ*_0_ = 1 ms, *n* = 8 and *β* = 2 corresponds to a frequency of about 4 600 Hz. By varying the parameters *σ*_0_ and *n*, we can move the frequency where deviations from true invariance begin to occur for a given value of the tolerance parameter *β*.

To prevent the temporal delay from being too long at low frequencies, one can also introduce a *soft upper bound on the temporal scale*

$$\begin{array}{c} \tau^{'}=\frac{\tau }{{\left(1+{\left(\frac{\tau }{\tau_{\infty }}\right)}^{p}\right)}^{1/p}} \end{array}$$ (75)

for suitable values of *τ*_∞_ and *p*. Then, approximate frequency covariance will hold over some subset of frequencies defined by the parameters *n*, *τ*_0_, *τ*_∞_ and *p*.

In human hearing, there is different evidence that the resolution of pitch perception is the highest in the area around 0.6-2 kHz and then decreases for both lower and higher frequencies (see e.g. Hartmann [65]; Moore [66]). Furthermore, within the middle area 0.6-2 kHz the relative pitch sensitivity appears to be approximately constant. The synchrony in the neural firing in the auditory nerve decreases with increasing frequency (Johnson [67]). The ability to identify the pitch of a mistuned harmonic decreases with increasing frequency exhibiting a knee at around 2 kHz (Hartmann et al. [68]). A lower frequency boundary can also be motivated from the size of the critical bands which according to the classic Zwicker data changes from being proportionally constant (about a musical minor third) for frequencies above 500 Hz to being constant (about 100 Hz) for frequencies below 500 Hz (Zwicker [69]). More recent data exhibit a similar but less strong tendency (Moore [70], page 77). Thus, in summary, there should presumable be both an upper and a lower limit for self-similarity.

## Receptive fields defined over the spectrogram

**Fig 9:**
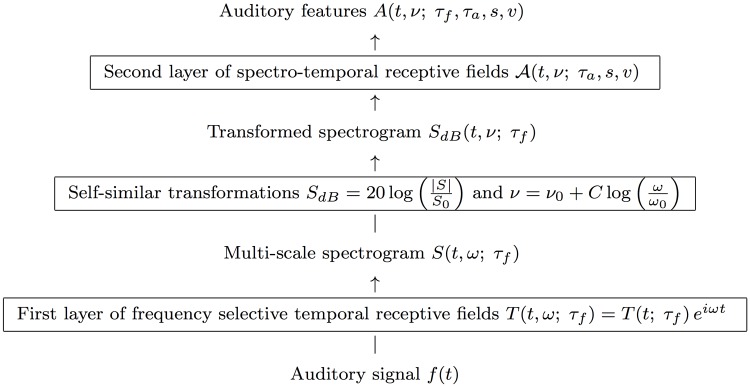
*Schematic illustration of the definition of auditory features from a second layer of receptive fields over the spectrogram*, where we also allow for a logarithmic transformation of the magnitude values ∣*S*∣ of the spectrogram prior to the application of the second layer of linear receptive fields and make use of a logarithmic transformation of the frequencies $\nu =\nu_{0}+C\log\left(\frac{\omega }{\omega_{0}}\right)$ before defining the linear receptive fields over the spectro-temporal domain. Regarding the scale parameters, the first layer of temporal receptive fields depends on a single temporal scale parameter *τ*_*f*_ for the frequency selective temporal filters, whereas the second layer of auditory receptive fields also depends on an additional temporal scale parameter *τ*_*a*_, a logspectral scale parameter *s* over the logarithmic frequencies *ν* and a glissando parameter *v* representing the rate by which the logarithmic frequencies may vary over time.

Given that a spectrogram has been computed by a first layer of auditory receptive fields, we define a *second layer of receptive fields* by operating on the spectrogram with 2-D spectro-temporal filters (see fig. 9) in a structurally similar way as visual receptive fields are applied to time-varying visual input (Lindeberg [5, 27]).

### Logarithmic transformations of the spectrogram

Prior to the definition of receptive fields from the spectrogram, it is natural to allow for a self-similar *logarithmic transformation of the magnitude values*

$$\begin{array}{c} S_{dB}=20\log_{10}\left(\frac{\left|S\right|}{S_{0}}\right). \end{array}$$ (76)

A logarithmic transformation of the magnitude of the spectrogram implies that a multiplicative transformation of the sound pressure *f* ↦ *a* *f*, corresponding to ∣*S*∣ ↦ *a*∣*S*∣, or an inversely proportional reduction in the sound pressure of the signal from a single auditory point source as function of distance *f* ↦ *f*/*R*, corresponding to ∣*S*∣ ↦ ∣*S*∣/*R*, are both transformed into a subtraction of the logarithmic magnitude by a constant

$$\begin{array}{c} \left|S\right|\mapsto \frac{a\;|S|}{R}\Rightarrow S_{dB}\mapsto S_{dB}+20\log_{10}a-20\log_{10}R. \end{array}$$ (77)

If we operate on the logarithmically transformed spectrogram by a receptive field 𝓐_Σ_ that is based on a combination of a spectro-temporal smoothing operation 𝓣_Σ_ with logspectral and temporal scale parameters as determined by a spectro-temporal covariance matrix Σ, temporal and/or logspectral derivatives ∂_*t*^*α*^*ν*^*β*^_ of orders *α* and *β* with at least one of *α* > 0 or *β* > 0

$$\begin{array}{c} {𝓐}_{\Sigma }\;S_{dB}=\partial_{t^{\alpha }\nu^{\beta }}{𝓣}_{\Sigma }\;S_{dB} \end{array}$$ (78)

then it follows that the influence on the receptive field responses of the constants *a* and *R*

$$\begin{array}{ccc} {𝓐}_{\Sigma }\;S_{dB} & = & \partial_{t^{\alpha }\nu^{\beta }}{𝓣}_{\Sigma }\;\left(S_{dB}+20\log_{10}a-20\log_{10}R\right)\\ & = & \partial_{t^{\alpha }\nu^{\beta }}{𝓣}_{\Sigma }\;S_{dB}+0+0 \end{array}$$ (79)

will be eliminated by the derivative operation if the constants *a* and *R* do not depend on time *t* or the logarithmic frequency *ν*, implying *invariance of the second-layer receptive field responses to variations in the sound pressure or the distance to a sound source*.

A logarithmic transformation of the magnitude is compatible with the *Weber-Fechner law*, stating that the ratio of an increment threshold Δ*I* of a stimulus for a just noticeable different relative to the background intensity *I* is constant over large ranges of magnitude variations, which approximately holds in both visual and auditory perception (Palmer [71]; Kandel et al. [72]).

Furthermore, since logarithmic frequencies constitute a natural metric for relating frequencies of sound (Fletscher [73]; Kandel et al. [72]; Young [74]) and there is an approximately logarithmic distribution of frequencies both on the basilar membrane (Greenwood [75]) and in the organization of the auditory cortex (Romani et al. [76]), it is natural to express these derived receptive fields in terms of *logarithmic frequencies*

$$\begin{array}{c} \nu =\nu_{0}+C\;\log\left(\frac{\omega }{\omega_{0}}\right) \end{array}$$ (80)

for some constants *C* and *ω*_0_, where specifically *ν*_0_ = 69, *C* = 12/log2 and *ω*_0_ = 2*π* ⋅ 440 correspond to logarithmic frequencies according to the MIDI standard.

This logarithmic parameterization of frequency implies that a shift in frequency, caused by *e.g*. transposing a piece of music by one octave, or varying the fundamental frequency in singing resulting in a multiplicative transformation of the harmonics (overtones), correspond to a mere *translation* in logarithmic frequency.

### Structural requirements on second-layer receptive fields

Given a transformed spectrogram defined in this way, let us define a family of second layer spectro-temporal receptive fields *A*(*t*, *ω*; Σ) that are to operate on the transformed spectrogram *S*_*dB*_(*t*, *ν*;*τ*) and be parameterized by some multi-dimensional spectro-temporal scale parameter Σ that includes smoothing over time *t* and logarithmic frequencies *ν*, and for which the corresponding operator 𝓐_Σ_ is required to obey:

(i) *linearity* over the logarithmic spectrogram
  $$\begin{array}{c} {𝓐}_{\Sigma }\left(a\;S_{1}+b\;S_{2}\right)=a{𝓐}_{\Sigma }\left(S_{1}\right)+b\;{𝓐}_{\Sigma }\left(S_{2}\right) \end{array}$$ (81)
  to ensure that (a) the multiplicative relations of the magnitude of the spectrogram (77) that are mapped to linear relations by the logarithmic transformation (76) are preserved as linear relations over the receptive field responses and (b) the scale-space properties imposed to ensure non-creation of new structures in smoothed spectrograms as defined by spectro-temporal smoothing kernels do also transfer to spectro-temporal derivatives of these,
(ii) *shift-invariance* with respect to translations over time *t* ↦ *t* + Δ*t* and logarithmic frequencies *ν* ↦ *ν* + Δ*ν*
  $$\begin{array}{c} {𝓐}_{\Sigma }\left({𝓢}_{\left(\Delta t,\Delta \nu \right)}S\right)={𝓢}_{\left(\Delta t,\Delta \nu \right)}\left({𝓐}_{\Sigma }S\right) \end{array}$$ (82)
  such that all temporal moments and all logarithmic frequencies are treated in a similar manner. Temporal shift invariance implies that an auditory stimulus should be perceived in a similar manner irrespective of when it occurs. Shift-invariance in the logarithmic frequency domain implies that, for example, a piece of music should be perceived in a similar manner if it is transposed by *e.g*. one octave.

These conditions together imply that the spectro-temporal receptive fields should be given by convolution with some two-dimensional kernel over the spectro-temporal domain (Hirschmann and Widder [41]):

$$\begin{array}{c} \left({𝓐}_{\Sigma }S_{dB}\right)\left(t,\nu ;\;\tau_{f},\Sigma \right)=\int_{\xi =-\infty }^{\infty }\int_{\eta =-\infty }^{\infty }T\left(\xi ,\eta ;\;\Sigma \right)\;S_{dB}\left(t-\xi ,\nu -\eta ;\;\tau_{f}\right)\;d\xi \;d\eta . \end{array}$$ (83)

To characterize what types of receptive fields are compatible with scale-space properties, we will next impose additional structural requirements, which will take different forms depending on whether the temporal dimension is treated in a time-causal or non-causal manner:

#### Relations between receptive fields at different spectro-temporal scales

For pre-recorded sound signals, for which we can take the freedom of accessing data from the virtual future in relation to any time moment, we impose a

(iii.a) continuous semi-group structure over spectro-temporal scales on the second layer receptive fields
  $$\begin{array}{c} T\left(\cdot ,\cdot ;\;\Sigma_{2}\right)=T\left(\cdot ,\cdot ;\;\Sigma_{2}-\Sigma_{1}\right)*T\left(\cdot ,\cdot ;\;\Sigma_{1}\right) \end{array}$$ (84)
  corresponding to an additive structure over the multi-dimensional scale parameter Σ.

For time-causal signals, we require:

(iii.b) a continuous semi-group structure over logspectral scales *s*
  $$\begin{array}{c} T\left(\cdot ;\;s_{2}\right)=T\left(\cdot ;\;s_{2}-s_{1}\right)*T\left(\cdot ;\;s_{1}\right) \end{array}$$ (85)
  and a Markov property between adjacent temporal scales
  $$\begin{array}{c} T\left(\cdot ;\;\tau_{k+1}\right)=\left(\Delta T\right)\left(\cdot ;\;k\right)*T\left(\cdot ;\;\tau_{k}\right). \end{array}$$ (86)

These requirements are analogous to the previous treatment in the section “Structural requirements on temporal receptive fields”, with extensions from a purely temporal domain to a spectro-temporal domain.

#### Non-creation of new spectro-temporal structures with increasing scale

When processing the spectrogram at different spectro-temporal scales, we want to ensure that the spectro-temporal receptive fields do not create new structures at coarser scales that do not correspond to simplifications of corresponding structures at finer scales. Depending on whether the temporal dimension is treated in a time-causal or non-causal manner, we formalize this condition as:

(iv.a) For the non-causal Gaussian spectrogram (54), for which temporal causality of the temporal smoothing kernels is disregarded, we require *non-enhancement of local extrema* in the sense that if for some scale Σ_0_ the point (*t*_0_, *ν*_0_) is a local maximum (minimum) for the mapping (*t*, *ν*) ↦ (𝓐_Σ_ *S*_*dB*_)(*t*, *ν*; Σ_0_) then the value at this point must not increase (decrease) with increasing scale Σ.
(iv.b) For the time-causal spectrogram (55) based on truncated exponential filters coupled in cascade (33), we require: (iv.b1) the smoothing operation over the logspectral domain to satisfy non-enhancement of local extrema in the sense that if at some logspectral scale *s*_0_ a point *ν*_0_ is a local maximum (minimum) of the mapping *ν* ↦ (𝓐_Σ_ *S*_*dB*_)(*ν*; *s*_0_) obtained by disregarding the temporal variations, then the value at this point must not increase (decrease) with increasing logspectral scale *s*, and (iv.b2) the smoothing operation over time to be a time-causal scale-space kernel guaranteeing non-creation of new local extrema under an increase of the temporal scale parameter *τ*.

#### Glissando covariance

In musical performance, the frequencies may vary continuously over time in such a way that the fundamental frequency *ω*_1_ and the harmonics (overtones) *ω*_*j*_ are all multiplied by the same time-varying factor *ω*_*j*_(*t*) = *ψ*(*t*)*ω*_*j*_. This is in particular prominent in singing, but may occur in all instruments with continuous pitch control. In terms of logarithmic frequencies, we can model a local linearization of this temporal variability as a *glissando transformation* of the form

$$\begin{array}{c} \nu \left(t\right)=\nu_{0}+v\;t \end{array}$$ (87)

analogous to the way spatial image data may be subject to local Galilean transformations over time. Comparing two spectrograms, one with constant frequencies over time and one with linearly varying logarithmic frequencies, the glissando transformation can be expressed in operator form as

$$\begin{array}{c} S^{'}={𝒢}_{v}\;S\;\;\text{corresponding}\;\text{to}\;\;S^{'}\left(t,\nu^{'}\right)=S\left(t,\nu \right) \end{array}$$ (88)

for *ν*′ = *ν* + *v* *t*. In relation to receptive field responses that are computed over the two domains with spectro-temporal scale parameters Σ and Σ′, we may require:

(v) If two local patches of two spectrograms are related by a local glissando transformation, then it should be possible to relate the local spectro-temporal receptive field responses such that
  $$\begin{array}{c} {𝓐}_{G_{v}\left(\Sigma \right)}\;{𝒢}_{v}\;S={𝒢}_{v}\;{𝓐}_{\Sigma }\;S \end{array}$$ (89)
  for some transformation Σ′ = *G*_*v*_(Σ) of the spectro-temporal scale parameters Σ.

### Idealized models for spectro-temporal receptive fields

Given the structural requirements above, it can from derivations similar to those that are used for constraining visual receptive fields given structural requirements on a visual front-end (Lindeberg [5]) be shown that the second layer of auditory receptive fields should be based on spectro-temporal receptive fields of the form

$$\begin{array}{c} A\left(t,\nu ;\;\Sigma \right)=\partial_{t^{\alpha }\nu^{\beta }}\left(g\left(\nu -vt;\;s\right)\;T\left(t;\;\tau_{a}\right)\right) \end{array}$$ (90)

where

- ∂_*t*^*α*^_ represents a *temporal derivative operator* of order *α* with respect to time *t* which could alternatively be replaced by a glissando-adapted temporal derivative of the form $\partial_{\bar{t}}=\partial_{t}+v\;\partial_{\nu }$,
- ∂_*ν*^*β*^_ represents a *logspectral derivative operator* of order *β* with respect to logarithmic frequency *ν*,
- *T*(*t*; *τ*_*a*_) represents a *temporal smoothing kernel* with temporal scale parameter *τ*_*a*_, which should either be (i) a temporal Gaussian kernel *g*(*t*; *τ*_*a*_) of the form (26) or (ii) the equivalent time-causal kernel *h*_*composed*_(*t*; *μ*) according to (34) and corresponding to a set of truncated exponential kernels coupled in cascade,
- *g*(*ν* − *vt*; *s*) represents a Gaussian *spectral smoothing kernel* over logarithmic frequencies *ν* with logspectral scale parameter *s* and *v* representing a glissando parameter making it possible to adapt the receptive fields to variations in frequency *ν*′ = *ν* + *vt* over time, and
- the spectro-temporal covariance matrix Σ in the left hand side expression for spectro-temporal receptive fields comprises both the temporal scale parameter *τ*_*a*_, the logspectral scale parameter *s* and the glissando parameter *v*.

Thereby, the spectro-temporal receptive fields according to (90) constitute a combination of the purely temporal receptive fields according to the theory in the sections “Structural requirements on temporal receptive fields” and “Scale-space concepts for purely temporal domains” with a Gaussian scale-space concept over the logspectral dimension.

The proofs concerning these spectro-temporal receptive fields are similar to those regarding the spatio-temporal receptive fields over a 1+1-D spatio-temporal domain with the spatial dimension replaced by a logspectral dimension.

**Fig 10:**
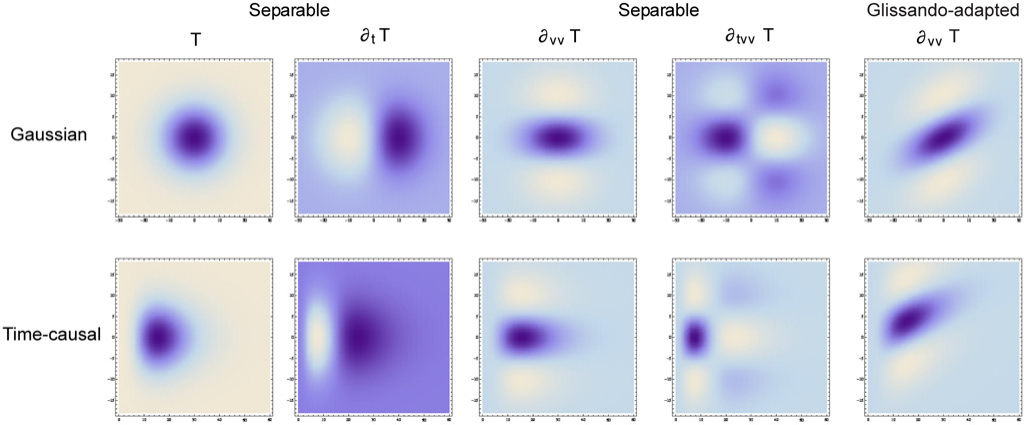
*Examples of idealized spectro-temporal receptive fields as obtained from spectro-temporal derivatives of spectro-temporal smoothing kernels* based on (top row) the non-causal Gaussian scale-space concept and (bottom row) the time-causal scale-space concept corresponding to first-order integrators coupled in cascade (here using five temporal scale levels and $c=\sqrt{2}$). The first four columns show separable receptive fields whereas the fifth column shows non-separable glissando-adapted receptive fields. (Horizontal dimension: time *t* in ms. Vertical dimension: Logarithmic frequency: *ν*. Temporal scale: $\sigma_{t}=\sqrt{\tau }=10\;\text{ms}$. Logspectral scale: $\sigma_{\nu }=\sqrt{s}=6$ semitones.)

Fig. 10 shows examples of spectro-temporal receptive fields obtained in this way for the two different types of underlying temporal scale-space concepts. For *ν* = 0, the resulting receptive fields are separable over the spectro-temporal domain, whereas *ν* ≠ 0 leads to non-separable glissando-adapted receptive fields.

#### Filter parameters of auditory receptive fields

The auditory features computed from these types of receptive fields depend on three different *scale parameters*:

- a *temporal window scale* parameter *τ*_*f*_ defining the temporal extent of the windows over which the windowed Fourier transforms in the spectrograms are defined,
- a secondary *temporal integration scale* parameter *τ*_*a*_ defining the temporal extent over which the magnitude values in the spectrogram are integrated over time and
- a *logspectral scale* parameter *s* defining the amount of smoothing over logarithmic frequencies *ν*.

In addition, this family of spectro-temporal receptive fields comprises:

- a *glissando parameter* *v* that makes it possible to adapt the receptive fields to variations on the logarithmic frequencies *ν* over time *t*.

Each parameterized spectro-temporal receptive field may occur for different orders of differentiation *α* and *β* with respect to time and logarithmic frequencies, respectively.

### Auditory features from second layer receptive fields

In the following, we will show examples of auditory features that can be defined from a second layer of auditory receptive fields of this form.

#### Spectro-temporal smoothing

Auditory receptive fields *A* based on convolution with a spectro-temporal smoothing kernel *T* over the spectro-temporal domain:

$$\begin{array}{c} A\left(t,\nu ;\;\tau_{a},s,v\right)=T\left(t,\nu ;\;\tau_{a},s,v\right). \end{array}$$ (91)

#### Onset and offset detection

Computation of first-order temporal derivatives

$$\begin{array}{c} {𝒟}_{t}\left(t,\nu ;\;\tau_{a},s\right)=\sqrt{\tau_{a}}\;\partial_{t}T\left(t,\nu ;\;\tau_{a},s\right) \end{array}$$ (92)

where $\sqrt{\tau_{a}}$ is a scale normalization factor to approximate *scale-normalized derivatives* (Lindeberg [77]) by variance normalization (Lindeberg and Bretzner [78]).

**Fig 11:**
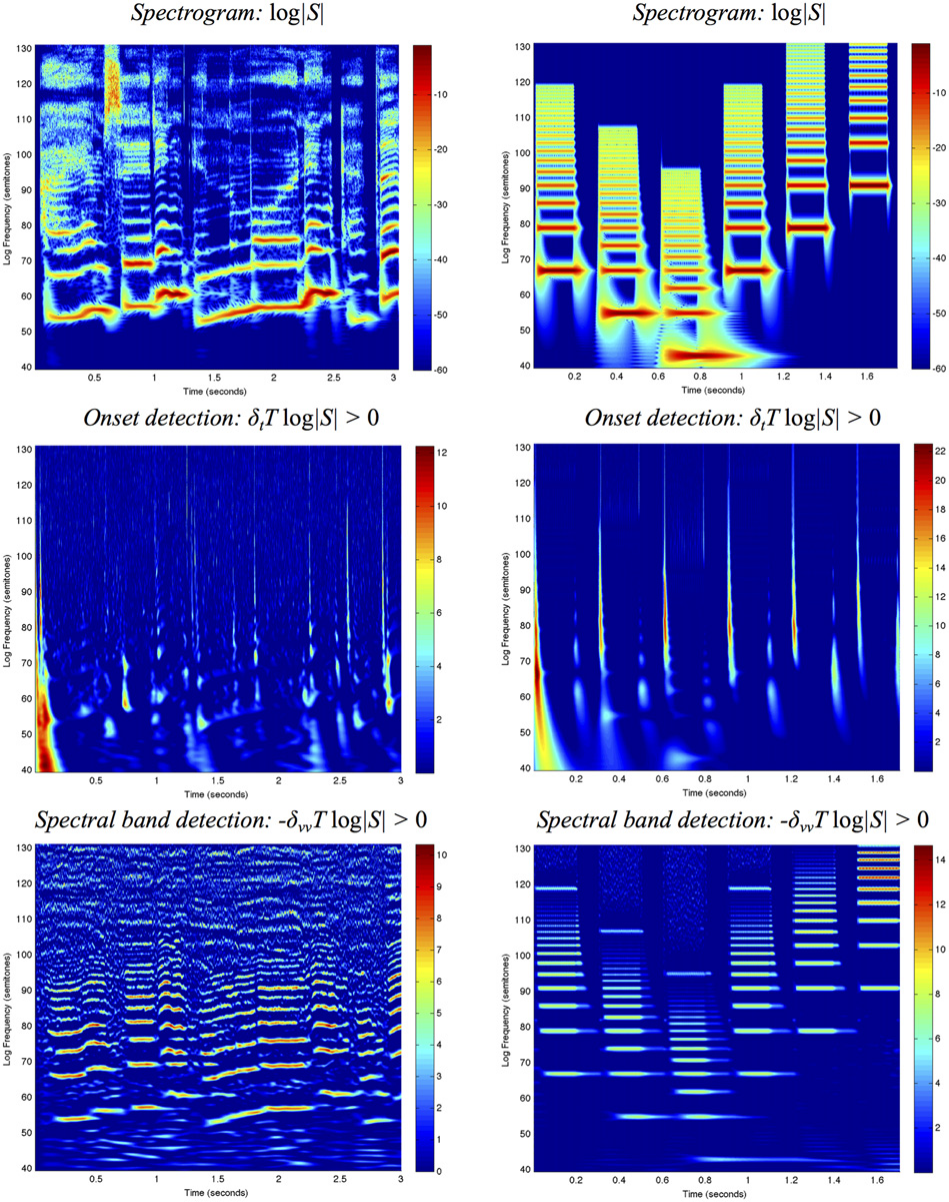
*Second layer receptive field responses obtained by applying spectro-temporal scale-space derivatives to the logarithmic spectrogram* of (left column) the first 3 seconds of “Tom’s diner” by Suzanne Vega with the lyrics “I am sitting in the morning at the …” or (right column) a synthetic signal containing of harmonic spectra with 20 partial tones and a spectral slope of 6 dB/octave at different fundamental frequencies: (top row) Original spectrogram. (middle row) *Onset detection* from the positive part of the first-order temporal derivatives ∂_*t*_ 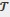 log ∣*S*∣ > 0 using a cascade of four time-recursive filters with temporal scale proportional to the temporal window scale $\sigma_{t}=\sqrt{\tau }=0.75\;\sigma_{w}$ and with logspectral smoothing scale *σ*_*ν*_ = 0.5 semitones. (bottom row) *Spectral band detection* from the negative part of the second-order temporal derivatives ∂_*νν*_ 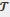 log ∣*S*∣ < 0. The vertical axis shows the logarithmic frequency expressed in semitones with 69 corresponding to the tone A4 (440 Hz).

This operation is similar to edge detection in image processing and computer vision (Canny [79]; Lindeberg [80]) with the differences that (i) the underlying derivatives are computed in a fixed direction and that (ii) in the case of a time-causal treatment of time, the onset detection will also be associated with a temporal delay. The signed derivative operator responds to an increase in the magnitude of the signal by a positive response and to a decrease in the magnitude by a negative response. To select receptive field responses that correspond to onsets only, this operation is naturally combined with the (non-linear) logical operation: *D*_*t*_ > 0 such that (see fig. 11, middle row)

$$\begin{array}{c} {𝓐}_{onset}\;S_{dB}=\left\{\begin{array}{cc} {𝒟}_{t}\;S_{dB} & \text{if}\;{𝒟}_{t}\;S_{dB}>0\\ 0 & \text{otherwise} \end{array}\right. \end{array}$$ (93)

Analogously, offset detection can be performed using

$$\begin{array}{c} {𝓐}_{offset}\;S_{dB}=\left\{\begin{array}{cc} -{𝒟}_{t}\;S_{dB} & \text{if}\;{𝒟}_{t}\;S_{dB}<0\\ 0 & \text{otherwise} \end{array}\right. \end{array}$$ (94)

#### Spectral sharpening

Computation of second-order Gaussian derivatives over the logspectral domain

$$\begin{array}{c} {𝒟}_{\nu \nu }\left(t,\nu ;\;\tau_{a},s\right)=s\;\partial_{\nu \nu }T\left(t,\nu ;\;\tau_{a},s\right) \end{array}$$ (95)

where the factor *s* is a scale normalization factor for scale-normalized derivatives based on the Gaussian scale-space concept (Lindeberg [77]). Depending on the value of the logspectral scale parameter, this operation may either enhance partial tones or formants. This operation is naturally combined with the (non-linear) logical operation 𝒟_*νν*_ < 0 (see fig. 11, bottom row)

$$\begin{array}{c} {𝓐}_{band}\;S_{dB}=\left\{\begin{array}{cc} -{𝒟}_{\nu \nu }\;S_{dB} & \text{if}\;{𝒟}_{\nu \nu }\;S_{dB}<0\\ 0 & \text{otherwise} \end{array}\right. \end{array}$$ (96)

**Fig 12:**
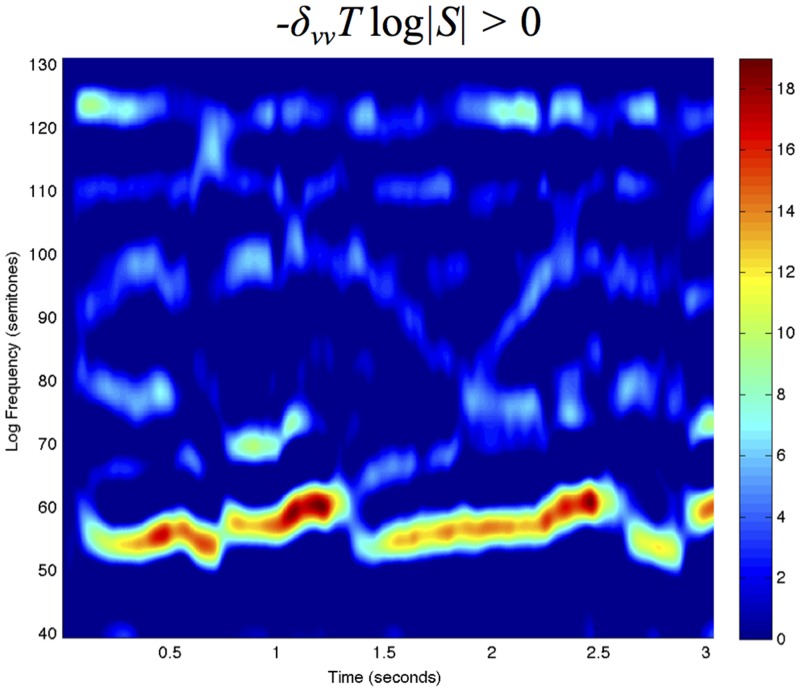
*Spectral sharpening at a coarse logspectral scale* (*σ*_*ν*_ = 4 semitones) applied to the spectrogram of the first 3 seconds of “Tom’s diner” with the lyrics “I am sitting in the morning at the …” using recursive filters at composed temporal scale 20 ms. Note how this operation *reveals the formants of the vowels* in the frequency range between roughly MIDI 70 and MIDI 110, corresponding to frequencies beween roughly 450 Hz and 4.7 kHz. The vertical axis shows the logarithmic frequency expressed in semitones with 69 corresponding to the tone A4 (440 Hz).

When applied at a *fine logspectral scale*, this operation can be used for enhancing *spectral bands* corresponding to the fundamental frequency and its overtones (see fig. 11, bottom row). When applied at a *coarser logspectral scale*, corresponding spectral sharpening can be used for enhancing the *formants of vowels* (see fig. 12). A similar approach involving a combination of Gaussian functions was used by Baer et al. [81] for enhancing spectral contrast for listeners with hearing impairment and by Heckmann et al. [55] as a part of feature extraction for automatic speech recognition.

By comparing the responses of the partial tones in the second-order logspectral derivatives to the partial tones in the raw logarithmic spectrogram, we can note that the responses to the partial tones are far more similar between different partial tones in the logspectral derivatives compared to the raw spectrogram. This property can be understood from the invariance of spectro-temporal derivatives to local multiplications of the magnitude of a signal described in the section “Logarithmic transformations of the spectrogram”. If we model the partial tones as self-similar copies of each other at different frequencies while having different relative strength (sound pressure), then by combination of the invariance under multiplications of the magnitude in the section “Logarithmic transformations of the spectrogram” with the invariance of the relative bandwidth under multiplicative frequency transformations in the section “Frequency selectivity of the spectrograms”, it follows that the spectro-temporal derivative responses to different overtones can be expected to have a similar appearance.

**Fig 13:**
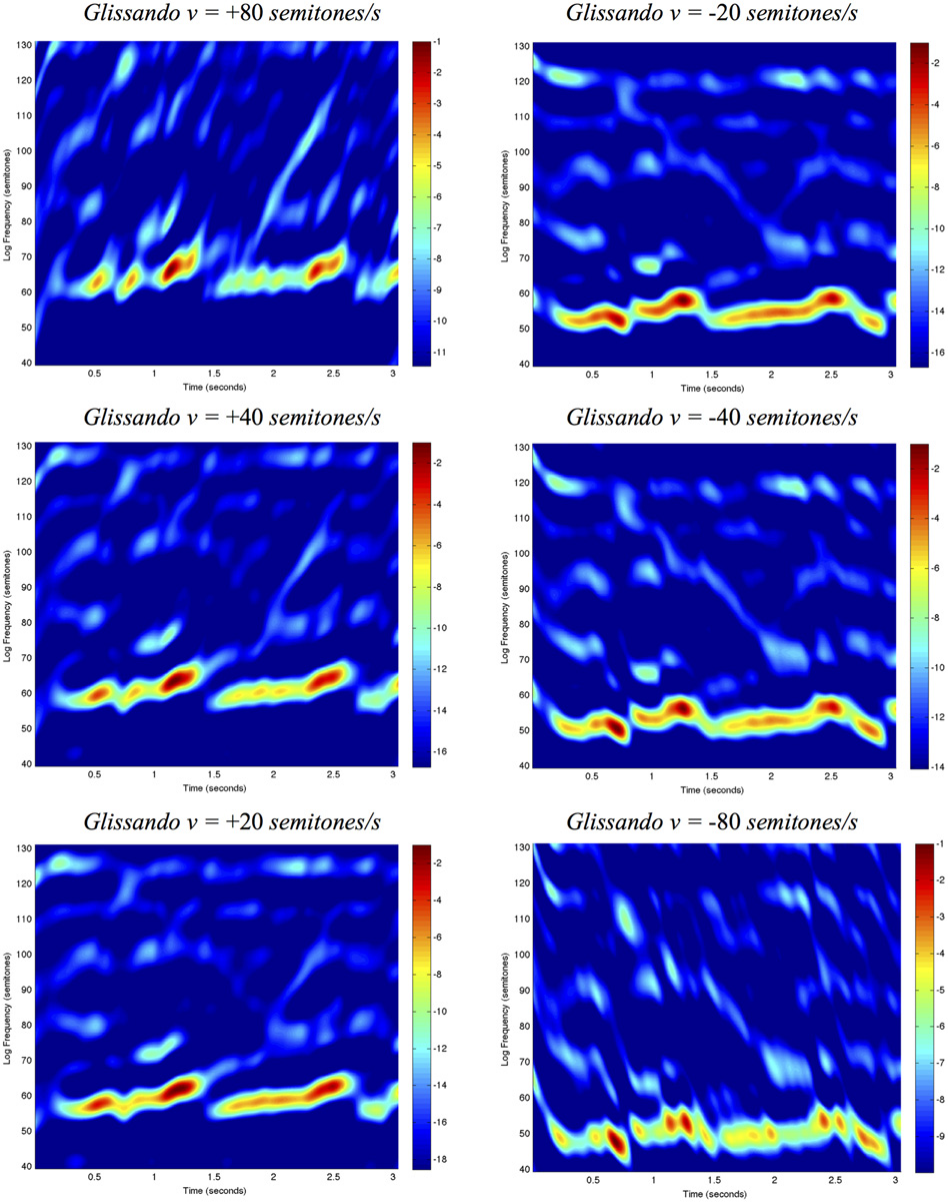
*Enhancement of the formants using glissando-adapted receptive fields* corresponding to second-order derivatives with respect to logarithmic frequency *ν* for different glissando values *v* = +80, +40, 20, -20, -40 and -80 and applied to the first 3 seconds of “Tom’s diner” computed with time-causal receptive fields at temporal scale *σ*_*t*_ = 20 ms and logspectral scale *σ*_*ν*_ = 4 semitones (compare with fig. 12 that shows corresponding results for non-adapted separable receptive fields). Note how the formant variations for different amounts of glissando are enhanced by glissando-adapted receptive fields for corresponding values of the glissando parameter. Such a set of glissando-adapted receptive fields for a logarithmic distribution of the glissando values *v* can serve as a filter bank for algorithms that operate on these receptive field responses as input. (Horizontal dimension: time *t*, Vertical dimension: logarithmic frequency *ν*.)

Fig. 13 shows an extension of this approach, where formant enhancement is performed using glissando-adapted receptive fields, demonstrating how formant variations for different amounts of glissando are enhanced by glissando-adapted receptive fields for corresponding values of the glissando parameter.

#### Capturing frequency variations over time

Given that local spectral bands have been enhanced by second-order derivatives over logarithmic frequencies (95), we can compute local extrema over frequencies by differentiating this response

$$\begin{array}{c} \partial_{\nu }\left(-{𝒟}_{\nu \nu }\;S_{dB}\right)=0, \end{array}$$ (97)

$$\begin{array}{c} \partial_{\nu \nu }\left(-{𝒟}_{\nu \nu }\;S_{dB}\right)<0. \end{array}$$ (98)

**Fig 14:**
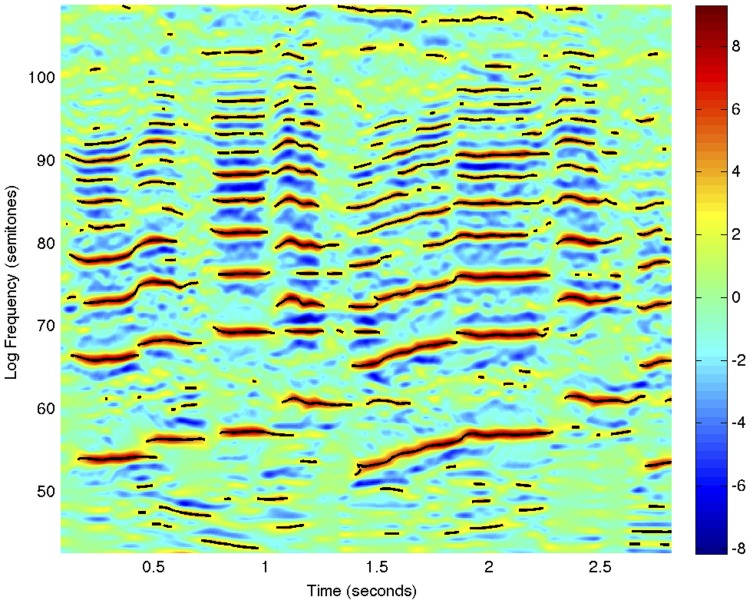
*Spectro-temporal curves that make explicit how the frequencies of partial tones vary over time*, computed as the zero-crossing curves of the logspectral derivative ∂_*ν*_(−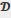
_*νν*_ log ∣*S*∣) = 0 that satisfy ∂_*νν*_(−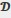
_*νν*_ log ∣*S*∣) < 0 and which thereby become continuous curves over time (drawn in black, thresholded on −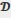
_*νν*_ log ∣*S*∣ ≥ *C* for *C* = 3 and overlaid on −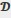
_*νν*_ log ∣*S*∣). (Horizontal dimension: time *t*, Vertical dimension: logarithmic frequency *ν*.)

By interpolating for the zero-crossings of (97) that satisfy the sign constraint (98) we can obtain subresolution *curves of how the frequency of partial tones vary over time* (see fig. 14).

#### Glissando estimation

One way to estimate explicitly how the frequencies vary over time is by estimating the temporal variation in the above curves, corresponding to feature tracking in the area of computer vision.

An alternative more receptive field based approach is by computing the receptive field responses for a *filter bank* of different glissando-receptive fields 𝒟(*v*) (*e.g*. second-order logspectral derivatives 𝒟(*v*) = 𝒟_*νν*_(*v*)) for different amounts of glissando *v* analogous to how ridge detection methods in computer vision can be expressed in terms of second-order derivatives of image intensity (Lindeberg [80]) and selecting the maximum response over the filter bank

$$\begin{array}{c} {𝓐}_{\Sigma }\;S_{db}=\max_{v}𝒟\left(v\right)\;S_{dB} \end{array}$$ (99)

as the glissando estimate

$$\begin{array}{c} \hat{v}={\text{argmax}}_{v}𝒟\left(v\right)\;S_{db} \end{array}$$ (100)

preferably complemented by interpolation to estimate the amount of glissando by higher accuracy than the actual sampling (compare with Laptev and Lindeberg [82] and Lindeberg [27] for corresponding filter-based approaches for estimating image velocities using a filter bank approach over different Galilean transformations).

Yet a more direct approach can be obtained by computing a *spectro-temporal second-moment matrix*

$$\begin{array}{ccc} ϒ(x,y;\;t,s) & = & \left(\begin{array}{cc} {ϒ}_{tt} & {ϒ}_{t\nu }\\ {ϒ}_{t\nu } & {ϒ}_{\nu \nu } \end{array}\right)\\ & = & \int_{\left(\xi ,\eta \right)\in {\mathbb{R}}^{2}}\left(\begin{array}{cc} L_{t}^{2} & L_{t}\;L_{\nu }\\ L_{t}\;L_{\nu } & L_{\nu }^{2} \end{array}\right)T\left(t-\xi ,\nu -\eta ;\;\tau ,s\right)\;d\xi \;d\eta \end{array}$$ (101)

by a third layer of spectro-temporal smoothing applied to the products $L_{t}^{2}$, *L*_*t*_ *L*_*ν*_ and $L_{\nu }^{2}$ of the spectro-temporal derivatives *L*_*t*_ = ∂_*t*_𝓣_Σ_ *S*_*db*_ and *L*_*ν*_ = ∂_*ν*_𝓣_Σ_ *S*_*db*_ and then computing the glissando estimate as the value

$$\begin{array}{c} v=-\frac{{ϒ}_{t\nu }}{{ϒ}_{\nu \nu }} \end{array}$$ (102)

that transforms the spectro-temporal moment matrix to diagonal form with the mixed ${ϒ}_{t\nu }^{\prime }$ being zero and corresponding to estimation of optic flow and Galilean invariant image descriptors in the area of computer vision (Lukas and Kanade [83]; Laptev et al. [84]; Lindeberg [27]). Specifically, by computing receptive field responses using a glissando estimate according to (100) alternatively for a glissando value that corresponds to a fixed-point of (102), it can be shown that *the resulting glissando-adapted receptive field responses will be invariant under glissando transformations*, which would not be fully possibly based on separable spectro-temporal receptive fields only (see also Lindeberg [5, 27] for analogous results regarding Galilean invariance in vision).

## Relations to biological receptive fields

**Fig 15:**
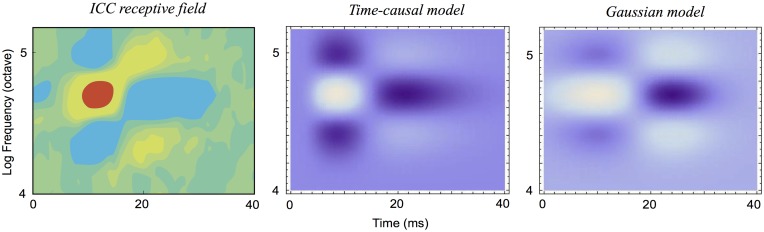
(left) *A separable monaural spectro-temporal receptive field in the central nucleus of the inferior colliculus (ICC) of cat* as reported by Qiu et al. [31] (schematic simplification only, see fig. 2(a) in [31] or fig. 16 in [64] for the original data). (middle and right) Idealized receptive fields models (90) corresponding to a first-order derivative with respect to time and a second-order derivative with respect to logarithmic frequency centered at *ν* = 4.7 octave, temporal scale *σ*_*t*_ = 7 ms, logspectral scale *σ*_*ν*_ = 0.17 octave for both models and additionally temporal delay *δ* = 17 ms for the Gaussian model.

**Fig 16:**
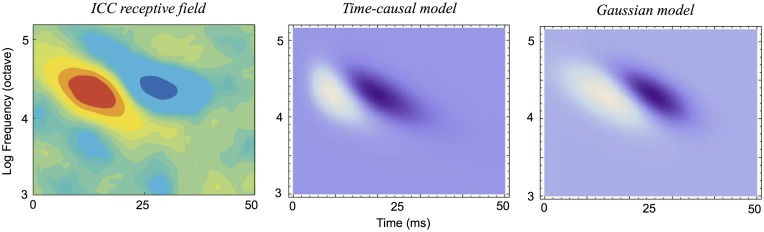
(left) *A non-separable spectro-temporal receptive field in the central nucleus of the inferior colliculus (ICC) of cat* as reported by Qiu et al. [31] (schematic simplification only, see fig. 3(a) in [31] or fig. 17 in [64] for the original data). (middle and right) First-order temporal derivative of idealized glissando-adapted receptive fields models (90) centered at *ν* = 4.3 octave, temporal scale *σ*_*t*_ = 7 ms, logspectral scale *σ*_*ν*_ = 0.20 octave and glissando *v* = −0.02 octave/ms for both models and additionally temporal delay *δ* = 23 ms for the Gaussian model.

**Fig 17:**
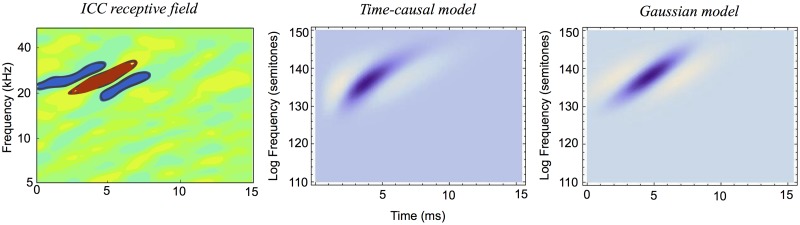
(left) *A non-separable spectro-temporal receptive fields in the inferior colliculus (ICC) of Mexican free-tailed bat* as reported by Andoni et al. [38] (schematic simplification only, see fig. 6(c) in [38] or fig. 18 in [64] for the original data). (middle and right) Second-order temporal derivative of idealized glissando-adapted receptive fields models (90) centered at semitone *ν* = 138, temporal scale *σ*_*t*_ = 2 ms, logspectral scale *σ*_*ν*_ = 3 semitones and glissando *v* = 1.5 semitones/ms for both models and additionally temporal delay *δ* = 4.7 ms for the Gaussian model.

In the central nucleus of the inferior colliculus (ICC) of cats, Qiu et al. [31] report that about 60% of the neurons can be described as separable in the time-frequency domain (see fig. 15), whereas the remaining neurons are either obliquely oriented (see fig. 16) or contain multiple excitatory/inhibitory subfields. Glissando-adapted receptive fields have also been reported in the inferior colliculus of bats (see fig. 17). This overall structure is nicely compatible with the treatment in the section “Idealized models for spectro-temporal receptive fields”, where the second-layer receptive fields are expressed in terms of spectro-temporal derivatives of either time-frequency separable spectro-temporal smoothing operations or corresponding glissando-adapted features as motivated by the structural requirements in the section “Structural requirements on second-layer receptive fields”.

**Fig 18:**
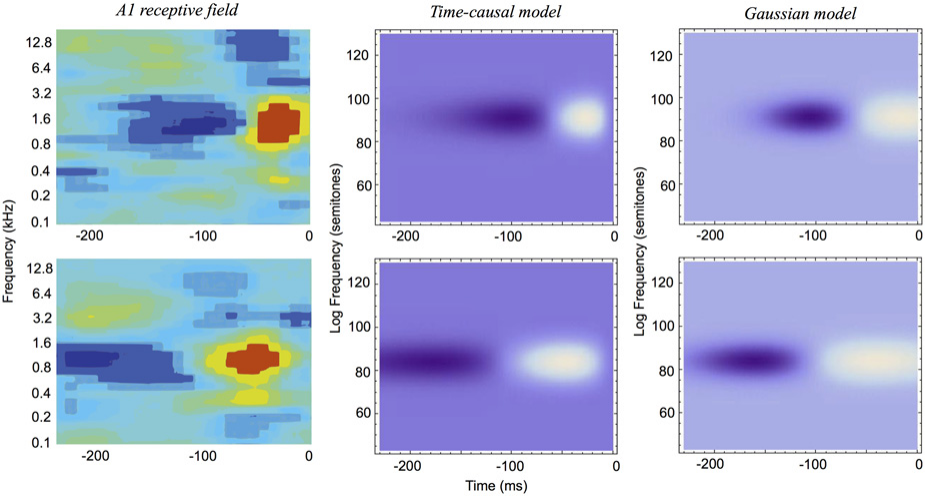
(left column) *Separable spectro-temporal receptive fields in the primary auditory cortex (A1) of Sprague Dawley rat* as reported by Machens et al. [37] (schematic simplification only, see figs. 5(a) and 5(c) in [37] or fig. 19 in [64] for the original data). (middle and right columns) Idealized receptive fields models (90) corresponding to first-order derivatives with respect to time, in the top row centered at semitone *ν* = 91, temporal scale *σ*_*t*_ = 45 ms, logspectral scale *σ*_*ν*_ = 6 semitones for both models and additionally temporal delay *δ* = 60 ms for the Gaussian model and in the bottom row centered at semitone *ν* = 84, temporal scale *σ*_*t*_ = 60 ms, logspectral scale *σ*_*ν*_ = 6 semitones for both models and additionally temporal delay *δ* = 100 ms for the Gaussian model

**Fig 19:**
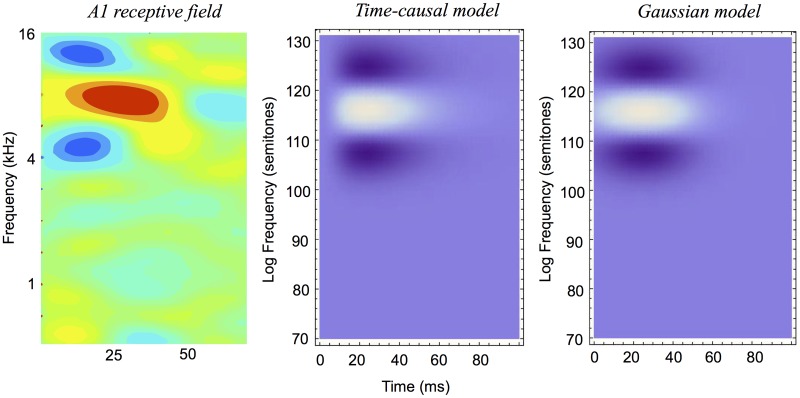
(left) *A separable spectro-temporal receptive fields in the primary auditory cortex (A1) of ferret* as reported by Elhilali et al. [39] (schematic simplification only, see fig. 6(a) in [39] or fig. 20 in [64] for the original data). (middle and right) Idealized receptive fields models (90) corresponding to second-order derivatives with respect to logarithmic frequency centered at semitone *ν* = 116, temporal scale *σ*_*t*_ = 17 ms, logspectral scale *σ*_*ν*_ = 5 semitones for both models and additionally temporal delay *δ* = 25 ms for the Gaussian model.

**Fig 20:**
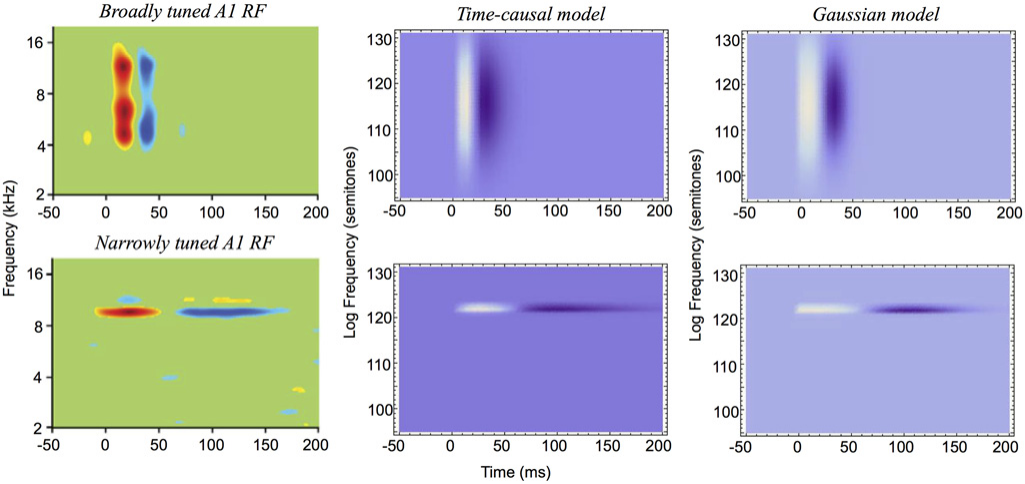
(left column) *Spectro-temporal receptive fields of broadly and narrowly tuned neurons in the primary auditory cortex (A1) of cats* as reported by Atencio and Schreiner [40]. In terms of the idealized models of receptive fields, such broad or narrowly tuned receptive fields correspond to different values of the logspectral scale parameter *s*. (middle and right columns) Idealized receptive fields models (90) corresponding to first-order derivatives with respect to time, in the left column centered at semitone *ν* = 119, temporal scale *σ*_*t*_ = 12 ms, logspectral scale *σ*_*ν*_ = 8 semitones for both models and additionally temporal delay *δ* = 20 ms for the Gaussian model and in the bottom column centered at semitone *ν* = 122, temporal scale *σ*_*t*_ = 45 ms, logspectral scale *σ*_*ν*_ = 0.5 semitones for both models and additionally temporal delay *δ* = 60 ms for the Gaussian model.

Qualitatively similar shapes of receptive fields can be measured from neurons in the primary auditory cortex (see figs. 18 and 19 as well as Miller et al. [3] regarding binaural receptive fields). Specifically, the use of multiple temporal and spectral scales as a main component in the model is in good agreement with biological receptive fields having different degrees of spectral tuning ranging from narrow to broad (see fig. 20) and different temporal extent (see fig. 18). Corresponding tradeoffs between spectral and temporal tuning occur in the inferior colliculus (Rodriguez et al. [85]). The distribution of latencies is, however, towards somewhat shorter latencies in the thalamus than in the auditory cortex (Miller et al. [3]) consistent with larger temporal scales in the auditory cortex than in the inferior colliculus.

Whereas spatio-temporal receptive fields estimated from neurons in the auditory cortex have been reported to reasonably well predict the neural responses for subsets of natural stimuli, Machens et al. [37] report that for many natural stimuli the responses of the auditory neurons in the primary auditory cortex cannot be predicted by the estimated linear receptive fields. Atencio et al. [86] also report that the dimensionality and thereby the variability of the receptive fields in the auditory cortex is significantly richer compared to the receptive fields in the inferior colliculus in the midbrain. Thus, the neurons in the primary auditory cortex appear to contain non-linearities whose functionality remains to be understood. In the inferior colliculus in the midbrain, Escabi and Schreiner [87] report that about 60% of the receptive fields can be well described in terms of linearly integrating neurons.

In the work by Qiu et al. [31], the measured biological receptive fields were fitted to Gabor functions as motivated by previous use of Gabor functions for modelling visual receptive fields (Marcelja [88]; Jones and Palmer [89, 90]). In vision, the use of Gabor functions for modelling visual receptive fields can, however, be questioned both on theoretical and empirical grounds (Stork and Wilson [91]; Lindeberg [5, 27]).

Stork and Wilson [91] argue that (i) only complex-valued Gabor functions, which cannot be described by single-unit receptive fields, minimize the uncertainty relation, (ii) the real functions that minimize this relation are Gaussian derivatives rather than Gabor functions and (iii) comparisons among Gabor and alternative fits to both psychophysical and physiological data have shown that in many cases other functions (including Gaussian derivatives) provide better fits than Gabor functions do.

Lindeberg [5, 27] argues that in relation to invariance properties the family of affine Gaussian kernels is closed under affine image deformations, whereas the family of Gabor functions obtained by multiplying rotationally symmetric Gaussians with sine and cosine waves is not closed under affine deformations. Therefore, it is not possibly to compute truly affine invariant representations from such Gabor functions. Instead, given a pair of images that are related by an affine image deformation, the lack of affine covariance implies that there will be a systematic bias in representations derived from such Gabor functions, corresponding to the difference between the backprojected Gabor functions in the two domains (compare with fig. 3 in [5] for an illustration). Using receptive profiles defined from directional derivatives of affine Gaussian kernels, it will on the other hand be possible to compute affine invariant representations. Similar arguments about Galilean invariance hold regarding theoretical modelling of spatio-temporal receptive fields.

In the visual domain, such affine transformations constitute a natural first-order model for image deformations caused by changing the viewing direction relative to an object or seeing an object that moves relative to the observer. In the auditory domain, affine transformations in terms of glissando transformations constitute a natural first-order model for frequencies that vary over time, such as in singing, melodic speech or sound from instruments having continuous pitch control.

Applied to the auditory domain, this means that invariance under glissando transformations cannot be accomplished with the regular family of Gabor functions, whereas glissando invariance is possible based on the presented theory for glissando-adapted receptive fields.

In addition, Gaussian receptive fiels as well as the derivatives of these can be modelled by diffusion equations, and can therefore be implemented by computations between neighbouring computational units, which is biologically plausible in terms of connections between neighbouring neurons and of minimizing the wiring length between them.

Specifically, biological spectro-temporal receptive fields show a marked temporal asymmetry that cannot be captured by Gabor functions for which the locations of excitatory and inhibitory subregions are uniformly spaced. Therefore, Qiu et al. [31] performed additional non-linear time warping to be able to fit the model to the data. Then, they modelled oblique receptive fields over the time-frequency domain using singular value decomposition to express any oblique receptive field as a sum of separable Gabor-based receptive fields.

By modelling the spatio-temporal receptive fields by a combination of time-causal scale-space kernels over time and Gaussian receptive fields over logarithmic frequencies, the temporal asymmetry of the kernels constitutes an integrated part of the theory, which cannot be accomplished by temporally symmetric smoothing operations as used in the Gabor model of receptive fields. Furthermore, oblique receptive fields in the time-frequency domain do also constitute an integrated part of the theory in terms of glissando transformations, and there is no need to decompose a glissando-receptive field as a sum of a possibly rather large number of spectro-temporal receptive fields, to be able to model the spectro-temporal receptive field in a quantitative manner. In addition, the model has been derived in a mathematically principled way from a set of structural requirements and the idealized receptive fields can be computed by a combination of diffusion equations and first-order integrators, and therefore by a biologically plausible neural architecture.

## Relations to previous work in audio processing

Previous auditory models of human hearing have to a large extent focused on the first stages of processing including the acoustical response of the cochlea and the following neurological responses in the auditory nerve. The auditory periphery is also the stage at which it was possible to collect the first physiological and neurological data. Recently, partly due to the fast technological progress in measurement techniques, models of more high-level functions in the auditory cortex begin to emerge (Meddis et al. [92]). Thus, the purpose has been to convert the incoming audio into a frequency-time representation in a similar way as is done in the physical-neural system in the cochlea. This is typically done in several stages taking into account both biological measurements and psychoacoustic listening test data.

The main stage is to simulate the physical resonance system in the cochlea. It is often implemented as filter bank in which the bandwidth and frequency position of each band is separated in a similar way as the cochlear nerves. A gammatone filter is often used since it has been show to be a reasonable approximation of the acoustic properties in the cochlea leading to the first neural input (Patterson et al. [93, 94]). Many other types of filters have been proposed, such as the gammachirp providing better fit to non-linearities with regard to the asymmetric frequency response at loud sound pressures (Irino and Patterson [58]; see also Chen et al. [95] and Lopez-Poveda and Meddis [96]). In a second stage, the response of the auditory nerves arriving from the inner hair cells in the cochlea are modelled. A common approach is to make a half-wave rectification, compression (square-root or logarithmic) and low-pass filtering (Patterson et al. [36]). In addition the local contrast can be enhanced both in time and frequency using an adaptive procedure (Patterson and Holdsworth [97]).

Previous computational toolboxes include the auditory image model (Aim-mat) by Bleek et al. [98]; see also Patterson et al. [99] which include all of the different parts above as well as some additional parts and the auditory model by Slaney [100].

Thus, these auditory image models are quite advanced and take into account a number of biological and perceptual phenomena. However, the biological data supporting these stages seems rather scarce in particular for complex sound signals such as music. The nerve responses have often been measured in animals rather than humans and with rather simple stimuli such as stationary sinusoids (Ruggero [101]). Many of the parts are modelled after psychoacoustic data again with simple stimuli and thus involving the whole auditory cortex and brain. A limitation is that advanced perceptual models can be adapted to closely model a certain type of perceptual data but will typically not extrapolate to other perceptual conditions. The recent loudness model by Chen et al. [95] closely approximates perceptual data concerning several aspects of loudness but applies only to steady-state sounds. This is an indication that the formulation of the underlying model(s) is still potentially open to alternative solutions. This is not surprising giving the complexity of the task and difficulties in obtaining biological data.

The traditional auditory models are not necessarily the best choice as a front-end for modelling more high-level perceptual aspects of music and speech. These models are often very demanding in terms of computer power and memory. In addition, the resulting data may not be suitable for further processing. Therefore, in practical applications, a front-end used for analyzing perceptual phenomena is often a simplification of the complete model. Recently, alternative models have been suggested which apply general principles of auditory perception but leaving out the detailed aspects. Such a model can be a good compromise between biological/perceptual reality and computational clarity and efficiency. For example, Chi et al. [102] used for the first stage 128 overlapping constant-Q bandpass filters with 24 filters/octave, a hair cell model with a high-pass filter, a non-linear compression, a membrane leakage low-pass filter, and a simplification of a lateral inhibitory network. In addition, they also modelled a second stage of cortical processing using spectro-temporal receptive fields (STRFs) applied on the spectrogram derived in the first stage (Chi et al. [102]). Another example of such a simplified two-stage model including an auditory spectrogram with STRFs applied to speech recognition was presented by Heckmanns et al. [55].

Traditionally, the short-time Fourier transform (STFT) has been used extensively for converting the signal to a time-frequency representation, presumably due to its efficient computer implementation, the fast Fourier transform (FFT). One of the major drawbacks with the STFT is the frequency resolution, which is constant in terms of Hertz. Since the ear is approximately logarithmic with regard to the frequency band distribution, a major part of the frequency data in the upper treble region is less relevant for a perceptual analysis. Similarly the resolution in the bass range is not enough for example for determining the musical pitch using a time window that can capture the onsets of fast notes (Muller et al. [103]).

One possibility to achieve a log-frequency spectrum is to use a bank of individual bandpass filters as discussed above and also in line with the currently proposed model. This can, however, be rather time consuming. An interesting computationally efficient compromise is therefore the constant-Q transform which uses traditional STFTs applied in a combination of downsampling and different time resolutions for different octaves (Brown and Puckette [104]). A computational toolbox was recently presented by Schörkhuber and Klapuri [105]. Using the constant-Q method, the frequency resolution will be the same across the spectrum. The time resolution will, however, vary significantly across the spectrum and still exhibit poor time resolution in the bass region.

Similar approaches using a second layer of receptive fields applied on the spectrogram have been used in particular in speech research using Gabor functions (Kleinschmidt [49]; Ezzat et al. [52]; Meyer and Kollmeier [106]; Heckmann et al. [55]; Wu et al. [32]). Heckmann et al. [55] used Gabor-based receptive fields of different orientations in the time-frequency plane of the spectrogram in combination with different transformations inspired by visual object recognition to capture the formant trajectories over time. The resulting features were shown to improve the performance of a speech recognition system in combination with traditional features such as mel frequency cepstral components (MFCC). In this article, we show how such and related auditory operations can be derived in principled manner.

## Summary and discussion

We have presented a theory for how idealized models of auditory receptive fields can be formulated based on structural constraints on the first stages of auditory processing. The theory includes (i) the definition of multi-scale spectrograms at different temporal scales in such a way that a spectrogram at any coarser temporal scale can be related to a corresponding spectrogram at any finer temporal scale using theoretically well-defined scale-space operations, and additionally (ii) how a second-layer of spectro-temporal receptive fields can be defined over a logarithmically transformed spectrogram in such a way that the resulting spectro-temporal receptive fields obey invariance or covariance properties under natural sound transformations including temporal shifts, variations in the sound pressure, the distance between the sound source and the observer, a shift in the frequencies of auditory stimuli or glissando transformations. Specifically, theoretical arguments have been presented showing how these idealized receptive fields are constrained to the presented forms from symmetry properties of the environment in combination with assumptions about the internal structure of auditory operations as motivated from requirements of handling different temporal and spectral scales in a theoretically well-founded manner.

By combining the scale-space approach with a local frequency analysis, we obtain a new way of deriving the Gabor filters as a complex-valued scale-space transform resulting from the Gaussian scale-space concept being applied to a temporal signal multiplied by a complex sine wave. We can also derive the Gamma-tone filters in a corresponding manner, as a time-causal complex scale-space transform obtained by applying a set of time-causal scale-space kernels based on first-order integrators with equal time constants coupled in cascade and applied to a temporal signal multiplied by a complex sine wave. In addition, the scale-space approach to multi-scale spectrograms leads to a new family of generalized Gamma-tone filters obtained from a logarithmic distribution of the intermediate temporal scales, and allowing for different trade-offs between filter characteristics such as frequency selectivity and temporal delay.

Then, given that a multi-scale spectrogram has been defined and transformed by taking the logarithm of the magnitude values and expressing the frequencies on a logarithmic frequency scale to ensure natural covariance properties under variations of the sound pressure or a frequency shift in the stimulus, the theory provides a second layer of receptive fields applied to the spectrogram, based on spectro-temporal derivatives of spectro-temporal scale-space kernels. We have shown how the derived models of idealized spectro-temporal receptive fields are uniquely determined given natural symmetry properties (scale-space axioms) and we have shown examples of how basic auditory features can be computed in this way.

Thus, the presented scale-space theory for auditory signals can be both related to existing models for auditory analysis and additionally leads to the formulation of a set of new models. Specifically, the presented theory provides a coherent framework by which auditory receptive fields at the first levels of processing in the auditory hierarchy can be expressed within the same theoretical framework. Moreover, the theory allows for provable invariance properties under temporal shifts, variations in sound pressure and logarithmic frequency shifts.

In relation to biology, the theoretical framework thus shows that idealized receptive field profiles can be derived by necessity given a set of idealized assumptions that reflect structural properties of the environment, and lead to predictions about receptive field profiles that are qualitatively very similar to receptive fields found by cell recordings in the inferior colliculus (ICC) and the primary auditory cortex (A1) of mammals. From this theoretical background, the auditory receptive fields in these auditory areas can therefore be seen as highly adapted to the structure of the environment and enabling the computation of invariant representations of the auditory input.

In relation to audio processing, the paper shows how one can derive three types of temporal filters for defining spectrograms, which provides a new way to derive Gabor filters and Gammatone filters in conceptually similar ways, and also to derive a new family of generalized Gammatone filters with additional degrees of freedom to obtain different trade-offs between the spectral selectivity and the temporal delay of time-causal filters. The paper also presents a theory for defining a second layer of receptive fields on such spectrograms and gives examples of how basic auditory features can be computed in this way. These results are derived by mathematical necessity from the assumptions. It is furthermore shown how such audio processing operations can be related to biological receptive fields in auditory perception as well as to a corresponding theory for visual receptive fields.

We propose that this theory should be of wide general interest for researchers in the audio processing community by providing theoretically well-founded and provably invariant/covariant audio operations for processing sound signals and to researchers working with computational modelling or measurements of receptive fields, auditory invariances, theoretical biology and psychophysics, by serving as a general theoretical foundation and understanding of how receptive fields in ICC and A1 support invariant visual processes at higher levels in the auditory hierarchy.

### Implications of the theory

Conceptually, the presented *normative theory of auditory receptive fields based on axiomatic scale-space theory for auditory signals* provides a coherent and unified model for how auditory receptive field profiles can be determined by necessity from structural properties of the environment and a synthesis of how invariance properties with respect to basic sound transformations is made possible by such receptive fields. Specifically, the theory explains how the basic types of linear receptive fields found in ICC and the linear component of receptive fields in A1 are constrained to their forms from the requirement that they (i) should have the ability to handle auditory structures at different temporal and spectral scales and (ii) enable the computation of invariant auditory representations under frequency shifts, temporal shifts, variations in sound pressure or the distance between the sound source and the observer as well as glissando transformations.

In relation to previous literature on measurements of auditory receptive fields in the inferior colliculus (ICC) and the primary auditory cortex (A1), as investigated by Miller et al. [3], Qiu et al. [31], Machens et al. [37], Andoni et al. [38], Elhilali et al. [39], Atencio and Schreiner [40] and others, the paper presents a theoretical model by which qualitatively similar types of receptive fields can be derived by necessity, based on a set of mathematical assumptions that reflect structural properties of the environment in combination with assumptions regarding the internal consistency between auditory receptive fields at different temporal and spectral scales.

In relation to the Gammatone (Johannesmaa [33]; Patterson et al. [34, 36]; Hewitt and Meddis [35]; Irino and Patterson [58]; Hohmann [60]; Schlute et al. [62]; Ngamkham et al. [63] and Gabor (Gabor [28]; Ezzat et al. [52]) approaches for defining spectrograms in audio processing, the paper shows how such filters can be derived by necessity from a small set of principled assumptions (scale-space axioms) and as well as how a new family of generalized Gammatone filters can be derived with larger flexibility between temporal and spectral properties.

In relation to Gabor approaches for defining a second layer of receptive fields on from the spectrogram (Kleinschmidt [49]; Ezzat et al. [52]; Meyer and Kollmeier [106]; Heckmann et al. [55]; Wu et al. [32]), the paper shows that there are theoretical arguments for preferring a combination of Gaussian and generalized Gammatone filters as a second layer of receptive fields. Specifically, such second-layer receptive fields ensure temporal causality and appropriate transformation properties under natural sound transformations.

### Future work

A scientifically highly interesting question would be to investigate quantitatively which one of the three families of idealized receptive fields leads to the best fit with biological data, the Gaussian model or either of the time-causal models based on either equal time constants in the first-order integrators or a self-similar distribution. To answer this question properly, one needs access to data from a sufficiently large and representative set of neurons, which we as authors do currently not have access to. For this paper, we have therefore instead made comparisons with receptive fields profiles as published in the open literature. The results reported in the section “Relations to biological receptive fields” show that our theory leads to predictions that are in good qualitative agreement with biological receptive fields as reported by several authors and for several animal species. To answer which one of the models agrees best with biological data, quantitative comparisons to raw data would be needed.

Such a quantitative investigation could also allow for empirical determination of the parameters in the parameterized receptive fields (temporal and spectral scale parameters, order of smoothing for the time-causal filters, glissando parameters and orders of temporal and spectral differentiation) as well as the distribution of these parameters over families of receptive fields.

Concerning limitations of the presented approach, we have in the present treatment defined the second layer of receptive fields from the magnitude values of the spectrogram only, thus ignoring the local phase information. A natural extension would be to extend the formulation of the second layer of receptive fields to include the local phase of the spectrogram, which for example may provide important cues to judge if partial tones may constitute components of a harmonic spectrum belonging to the same physical source, and to formulate binaural receptive fields that are sensitive to the three-dimensional volumes in auditory space where a stimulus occurs.

It should also be stressed that the present approach constitutes a linear and pure feed-forward model for local receptive fields, corresponding to constant values of the filter parameters in the local diffusion equations and recurrence relations that determine the formation of the receptive fields. An interesting extension would be to adapt these filter parameters to the local input data or using top-down information, which could then provide computational mechanisms to express stimulus- and/or task-dependent receptive fields as reported by Fritz et al. [4], Machens et al. [37], Elhilali et al. [39], Eggermont [107], David et al. [108] and Laudanski et al. [109] and furthermore to extend the use of local receptive fields centered around a single frequency to multi-local operations that combine information from several distinct frequencies (Pienkowski and Harrison [110]).

In relation to such more complex non-linear mechanisms, the presented linear theory can be seen as a first principled starting point that (i) enables the computation of basic auditory features for audio processing and (ii) generates predictions about basic receptive field profiles that are qualitatively similar to biological receptive fields as measured by cell recordings in the inferior colliculus (ICC) and the primary auditory cortex (A1).

## Frequency selectivity of the spectrograms

Consider a sine wave signal with angular frequency *ω*_0_:

$$\begin{array}{c} f\left(t\right)=\sin\omega_{0}t \end{array}$$ (103)

When computing the windowed spectrogram, we multiply this signal by sine and cosine waves of different angular frequencies *ω* and integrate by a window function *h*(*t*; *τ*) with temporal extent *τ*:

$$\begin{array}{c} c\left(t\right)=h\left(t;\;\tau \right)*\left(f(t)\cos\omega t)\right) \end{array}$$ (104)

$$\begin{array}{c} s\left(t\right)=h\left(t;\;\tau \right)*\left(f(t)\sin\omega t)\right) \end{array}$$ (105)

By the use of basic rules for trigonometric functions

$$\begin{array}{cc} f(t)\cos\omega t & =\sin\omega_{0}t\;\cos\omega t=\frac{1}{2}\left(-\sin\left(\omega -\omega_{0}\right)t+\sin\left(\omega +\omega_{0}\right)t\right) \end{array}$$ (106)

$$\begin{array}{cc} f(t)\sin\omega t & =\sin\omega_{0}t\;\sin\omega t=\frac{1}{2}\left(\cos\left(\omega -\omega_{0}\right)t-\cos\left(\omega +\omega_{0}\right)t\right) \end{array}$$ (107)

the result of convolving these components with the window function *h*(*t*; *τ*) can be expressed by multiplication with the Fourier transform $\hat{h}(\omega ;\;\tau )$:

$$\begin{array}{cc} c(t) & =\frac{1}{2}\left(-\hat{h}\left(\omega -\omega_{0};\;\tau \right)\;\sin\left(\omega -\omega_{0}\right)t+\hat{h}\left(\omega +\omega_{0};\;\tau \right)\;\sin\left(\omega +\omega_{0}\right)t\right) \end{array}$$ (108)

$$\begin{array}{cc} s(t) & =\frac{1}{2}\left(\hat{h}\left(\omega -\omega_{0};\;\tau \right)\;\cos\left(\omega -\omega_{0}\right)t-\hat{h}\left(\omega +\omega_{0};\;\tau \right)\;\cos\left(\omega +\omega_{0}\right)t\right) \end{array}$$ (109)

Concerning the magnitude of the spectrogram

$$\begin{array}{c} S\left(t\right)=\sqrt{c{\left(t\right)}^{2}+s{\left(t\right)}^{2}} \end{array}$$ (110)

it follows that

$$\begin{array}{ccc} S{\left(t\right)}^{2} & = & \frac{1}{4}\left(\hat{h}{\left(\omega -\omega_{0};\;\tau \right)}^{2}+\hat{h}{\left(\omega +\omega_{0};\;\tau \right)}^{2}\right.\\ & & \left.-\;2\cos\left(2\omega_{0}t\right)\;\hat{h}\left(\omega -\omega_{0};\;\tau \right)\;\hat{h}\left(\omega +\omega_{0};\;\tau \right)\right) \end{array}$$ (111)

Assuming that the window function *h* should be a low-pass filter, then for *ω* close to *ω*_0_ let us assume

$$\begin{array}{c} |\hat{h}\left(\omega -\omega_{0};\;\tau \right)\left|\gg \right|\hat{h}\left(\omega +\omega_{0};\;\tau \right)| \end{array}$$ (112)

Thereby, the dominant component of the spectrogram near *ω*_0_ will be given by

$$\begin{array}{c} S_{magn}\left(\omega ;\;\tau \right)\approx \frac{|\hat{h}\left(\omega -\omega_{0};\;\tau \right)|}{2} \end{array}$$ (113)

By normalizing this entity such that the maximum value at *ω* = *ω*_0_ equals one, we quantify the frequency selectivity for a frequency dependent window scale *τ*(*ω*) as

$$\begin{array}{c} R\left(\omega \right)=|\hat{h}\left(\omega -\omega_{0};\;\tau \left(\omega \right)\right)| \end{array}$$ (114)

which on a logarithmic dB scale assumes the form

$$\begin{array}{c} R_{dB}\left(\omega \right)=20\log_{10}\left|\hat{h}\left(\omega -\omega_{0};\;\tau \left(\omega \right)\right)\right| \end{array}$$ (115)

where we would ideally choose the temporal extent of the kernel in units of $\sigma =\sqrt{\tau }$ proportional to the wavelength *λ* = 2*π*/*ω* for any angular frequency *ω*:

$$\begin{array}{c} \tau \left(\omega \right)=\sigma^{2}={\left(n*\lambda \right)}^{2}={\left(\frac{2\pi n}{\omega }\right)}^{2} \end{array}$$ (116)

### 

#### Gaussian window functions

For a Gaussian window function we have

$$\begin{array}{c} \hat{g}\left(\omega ;\;\tau \right)=\int_{t=-\infty }^{\infty }g\left(t;\;\tau \right)\;e^{-i\omega t}dt=e^{-\omega^{2}\tau /2} \end{array}$$ (117)

With the temporal extent of the window function proportional to the wavelength for any frequency according to (116), the frequency selectivity is given by

$$\begin{array}{c} R_{gauss}\left(\omega \right)=e^{-\frac{2\pi^{2}n^{2}{\left(\omega -\omega_{0}\right)}^{2}}{\omega^{2}}} \end{array}$$ (118)

or in dB

$$\begin{array}{c} R_{dB,gauss}\left(\omega \right)=-\frac{40\pi^{2}n^{2}{\left(\omega -\omega_{0}\right)}^{2}}{\log10\;\omega^{2}} \end{array}$$ (119)

##### Window functions defined from cascade of truncated exponential functions

For the truncated exponential filters coupled in cascade, the Laplace transform is

$$\begin{array}{cc} H_{composed}\left(q;\;\mu \right) & =\int_{t=-\infty }^{\infty }\left({*}_{k=1}^{K}h_{exp}\left(t;\;\mu_{k}\right)\right)\;e^{-qt}\;dt=\prod_{k=1}^{K}\frac{1}{1+\mu_{k}q} \end{array}$$ (120)

implying that the Fourier transform is given by

$$\begin{array}{c} {\hat{h}}_{composed}\left(\omega ;\;\mu \right)=H_{composed}\left(i\omega ;\;\mu \right)=\prod_{k=1}^{K}\frac{1}{1+i\;\mu_{k}\;\omega } \end{array}$$ (121)

In the special case when all time constants *μ*_*k*_ are equal

$$\begin{array}{c} \mu_{k}=\sqrt{\frac{\tau }{K}} \end{array}$$ (122)

and with the temporal extent of the window function proportional to the wavelength according to (116), the frequency selectivity is given by

$$\begin{array}{cc} R_{rec-uni}\left(\omega \right) & =|{\hat{h}}_{composed}\left(\omega -\omega_{0};\;\tau \left(\omega \right),K\right)|=\frac{1}{{\left(1+\frac{4\pi^{2}n^{2}{\left(\omega -\omega_{0}\right)}^{2}}{K\omega^{2}}\right)}^{K/2}} \end{array}$$ (123)

or in dB

$$\begin{array}{c} R_{dB,rec-uni}\left(\omega \right)=-\frac{K}{2\log10}\log\left(1+\frac{4\pi^{2}n^{2}{\left(\omega -\omega_{0}\right)}^{2}}{K\omega^{2}}\right) \end{array}$$ (124)

In the special case when the intermediate temporal scale levels *τ*_*k*_ are instead distributed according to a logarithmic distribution with *τ*_*k*_ = *c* ^2(*k* − *K*)^ *τ* and *μ*_*k*_ according to (39) and (40), and with the temporal extent of the window function proportional to the wavelength according to (116), we obtain

$$\begin{array}{c} R_{rec-log}\left(\omega \right)=\frac{1}{\sqrt{1+\frac{4\pi^{2}c^{2(1-K)}n^{2}{\left(\omega -\omega_{0}\right)}^{2}}{\omega^{2}}}}\times \frac{1}{\prod_{k=2}^{K}\sqrt{1+\frac{4\pi^{2}c^{2(k-K-1)}\left(c^{2}-1\right)n^{2}{\left(\omega -\omega_{0}\right)}^{2}}{\omega^{2}}}} \end{array}$$ (125)

or in dB

$$\begin{array}{ccc} R_{dB,rec-log}\left(\omega \right) & = & -\frac{10}{\log10}\log\left(1+\frac{4\pi^{2}\;c^{2(1-K)}\;n^{2}{\left(\omega -\omega_{0}\right)}^{2}}{\omega^{2}}\right)\\ & & -\;\frac{10}{\log10}\sum_{k=2}^{K}\log\left(1+\frac{4\pi^{2}c^{2(k-K-1)}\;\left(c^{2}-1\right)\;n^{2}{\left(\omega -\omega_{0}\right)}^{2}}{\omega^{2}}\right) \end{array}$$ (126)

**Fig 21:**
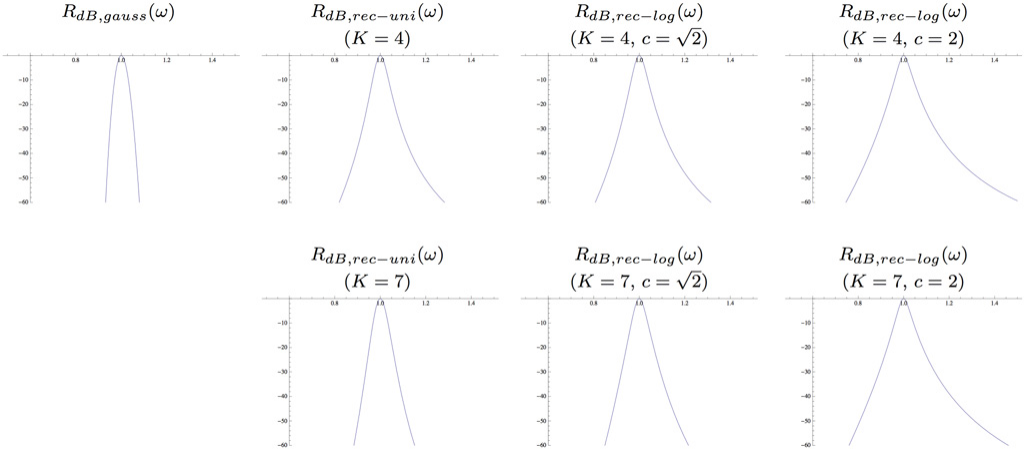
*Graphs of the frequency selectivities of Gaussian and time-causal window functions* with the temporal extent proportional to the corresponding wavelength of the spectrogram with *n* = 8. For the time-causal filters, *K* = 4 or *K* = 7 filters have been coupled in cascade. For the logarithmic distribution, the ratio between successive temporal scale levels *τ*_*k*_ = *c* ^2(*k* − *K*)^ *τ* has been determined from $c=\sqrt{2}$ or *c* = 2. (Horizontal axis: Angular frequency *ω* in units of *ω*_0_. Vertical axis: dB values down to -60 dB.)

Fig. 21 shows graphs of the frequency selectivity for the different types of temporal window functions and a few combinations of the underlying filter parameters. The non-causal Gaussian kernel has sharper frequency selectivity compared to the time-causal kernels. Within the class of time-causal kernels, the frequency selectivity increases with the number of truncated exponential kernels that are coupled in cascade. For the logarithmic distribution of the intermediate temporal scale levels, the frequency selectivity also increases with decreasing values of the distribution parameter *c*.

##### Dependency of the relative bandwidth on *n*

Notably all these expressions are functions of the ratio

$$\begin{array}{c} \frac{n^{2}{\left(\omega -\omega_{0}\right)}^{2}}{\omega^{2}}=\theta^{2} \end{array}$$ (127)

By for a fixed value of *θ* solving for *ω* and assuming *θ* > 0

$$\begin{array}{c} \omega_{1}=\frac{\omega_{0}}{1+\frac{\theta }{n}}\;\;\omega_{2}=\frac{\omega_{0}}{1-\frac{\theta }{n}} \end{array}$$ (128)

we get explicit expressions for how the relative bandwidth of the spectrogram corresponding to that specific value of *θ*

$$\begin{array}{cc} \frac{\omega_{2}-\omega_{1}}{\omega_{0}} & =\left(\frac{1}{1-\frac{\theta }{n}}-\frac{1}{1+\frac{\theta }{n}}\right)=\frac{\frac{2\theta }{n}}{1-{\left(\frac{\theta }{n}\right)}^{2}}\approx \frac{2\theta }{n}+𝒪\left({\left(\frac{\theta }{n}\right)}^{3}\right) \end{array}$$ (129)

alternatively in logarithmic MIDI units

$$\begin{array}{c} 12\log_{2}\left(\frac{\omega_{2}}{\omega_{1}}\right)=12\log_{2}\left(\frac{1+\frac{\theta }{n}}{1-\frac{\theta }{n}}\right)\approx \frac{24}{\log2}\frac{\theta }{n}+𝒪\left({\left(\frac{\theta }{n}\right)}^{3}\right) \end{array}$$ (130)

depends on *n* for any *θ*, implying that the relative bandwidth decreases approximately inversely proportional to *n*, where *θ* is related to the dB level *R*_*dB*_ < 0 according to

$$\begin{array}{c} \theta_{gauss}=\frac{\sqrt{\log10}}{2\pi }\sqrt{\frac{-R_{dB}}{10}} \end{array}$$ (131)

for the Gaussian window functions and according to

$$\begin{array}{c} \theta_{rec-uni}=\frac{\sqrt{K}}{2\pi }\sqrt{10^{-\frac{R_{dB}}{10K}}-1} \end{array}$$ (132)

for the time-causal kernels having a uniform distribution of the intermediate temporal scale levels. For the time-causal kernels having a logarithmic distribution of the intermediate scale levels, the parameter *θ* can be determined by solving the following equation

$$\begin{array}{ccc} R_{dB} & = & -\frac{10\log\left(4\pi^{2}\theta^{2}c^{2-2K}+1\right)}{\log(10)}\\ & & -\;\frac{20\sum_{k=2}^{K}\frac{1}{2}\log\left(4\pi^{2}\left(c^{2}-1\right)\theta^{2}c^{2k-2K-2}+1\right)}{\log(10)} \end{array}$$ (133)

numerically for specific values of *K* and *c*. Table 1 shows such values for *K* = 4 and *K* = 7 for $c=\sqrt{2}$ and *c* = 2 as well as corresponding values for a uniform distribution of the intermediate scale levels and a Gaussian window function.

**Table 1:** *Numerical values of the parameter θ determining the relative bandwidth* $\frac{2\theta }{n}$ *of the spectrogram* according to (129), for a Gaussian function and *K* truncated exponential kernels in cascade with a uniform distribution of the intermediate temporal scale levels *τ*_*k*_ = *τ*/*K* or a logarithmic distribution *τ*_*k*_ = *c* ^2(*k* − *K*)^ *τ* with *c* > 1.

Relative bandwidth of temporal window functions				
	-3 dB	-10 dB	-20 dB	-30 dB
*θ*_*gauss*_	0.132	0.242	0.342	0.418
*θ*_*rec* − *uni*_ (*K* = 4)	0.138	0.281	0.468	0.684
*θ*_*rec* − *log*_ ($K=4,c=\sqrt{2}$)	0.140	0.292	0.498	0.736
*θ*_*rec* − *log*_ (*K* = 4, *c* = 2^3/4^)	0.143	0.312	0.553	0.838
*θ*_*rec* − *log*_ (*K* = 4, *c* = 2)	0.146	0.332	0.619	0.971
*θ*_*rec* − *uni*_ (*K* = 7)	0.136	0.263	0.406	0.546
*θ*_*rec* − *log*_ ($K=7,c=\sqrt{2}$)	0.140	0.289	0.478	0.678
*θ*_*rec* − *log*_ (*K* = 7, *c* = 2^3/4^)	0.143	0.311	0.547	0.816
*θ*_*rec* − *log*_ (*K* = 7, *c* = 2)	0.146	0.332	0.617	0.963

##### Frequency invariance

From the invariance of the expressions (118), (123) and (125) under frequency transformations of the form

$$\begin{array}{c} \omega \mapsto \alpha \;\omega \end{array}$$ (134)

$$\begin{array}{c} \omega_{0}\mapsto \alpha \;\omega_{0} \end{array}$$ (135)

for any *α* > 0, it follows that the relative bandwidth of the spectrogram will be independent of the angular frequency *ω*. Thereby, over the range of frequencies for which the temporal extent of the window function is proportional to the wavelength, the spectral sensitivity will be invariant under a shift in frequency *ω* ↦ *α* *ω*, providing a foundation for frequency covariant receptive fields at higher levels in the auditory hierarchy.

## Temporal dynamics of the time-causal kernels

For the time-causal filters obtained by coupling truncated exponential kernels in cascade, there will be an inevitable temporal delay depending on the time constants *μ*_*k*_ of the individual filters.

A most straightforward way of estimating this delay is by using the additive property of mean values under convolution

$$\begin{array}{c} m=\sum_{k=1}^{K}\mu_{k} \end{array}$$ (136)

In the special case of all the time constants being equal $\mu_{k}=\sqrt{\tau /K}$, this measure is given by

$$\begin{array}{c} m_{uni}=\sqrt{K\tau }=\frac{2\pi \sqrt{K}n}{\omega } \end{array}$$ (137)

showing that the temporal delay increases if the temporal smoothing operation is divided into a larger number of smaller individual smoothing steps.

In the special case when the intermediate temporal scale levels are instead distributed logarithmically according to (38), with the individual time constants given by (39) and (40), this measure for the temporal delay is given by

$$\begin{array}{ccc} m_{log} & = & \frac{c^{-K}\left(c^{2}-\left(\sqrt{c^{2}-1}+1\right)c+\sqrt{c^{2}-1}c^{K}\right)}{c-1}\;\sqrt{\tau }\\ & = & \frac{2\pi n\;c^{-K}\left(c^{2}-\left(\sqrt{c^{2}-1}+1\right)c+\sqrt{c^{2}-1}c^{K}\right)}{(c-1)\;\omega } \end{array}$$ (138)

with the limit value

$$\begin{array}{c} m_{log-limit}=\lim_{K\to \infty }m_{log}=\frac{\sqrt{c^{2}-1}}{c-1}\sqrt{\tau }=\frac{\sqrt{c^{2}-1}}{c-1}\frac{2\pi n}{\omega } \end{array}$$ (139)

when the number of filters tends to infinity.

By comparing Equations (137), (138) and (139), we can specifically note that with increasing number of intermediate temporal scale levels, a logarithmic distribution of the intermediate scale levels implies shorter temporal delays than a uniform distribution of the intermediate scale levels.

Table 2 shows numerical values of these measures for different values of *K* and *c*. As can be seen from the table, the logarithmic distribution of the intermediate scales allows for significantly faster temporal dynamics than a uniform distribution.

**Table 2:** *Numerical values of the temporal delay in terms of the temporal mean* $m=\sum_{k=1}^{K}\mu_{k}$ *in units of* $\sigma =\sqrt{\tau }$ for time-causal kernels obtained by coupling *K* truncated exponential kernels in cascade in the cases of a uniform distribution of the intermediate temporal scale levels *τ*_*k*_ = *kτ*/*K* or a logarithmic distribution *τ*_*k*_ = *c* ^2(*k* − *K*)^ *τ* with *c* > 1.

Temporal mean values of time-causal kernels				
*K*	*m*_*uni*_	*m*_*log*_ ($c=\sqrt{2}$)	*m*_*log*_ (*c* = 2^3/4^)	*m*_*log*_ (*c* = 2)
2	1.414	1.414	1.399	1.366
3	1.732	1.707	1.636	1.549
4	2.000	1.914	1.777	1.641
5	2.236	2.061	1.860	1.686
6	2.449	2.164	1.910	1.709
7	2.646	2.237	1.940	1.721
8	2.828	2.289	1.957	1.732

### 

#### Additional temporal characteristics

Because of the asymmetric tails of the time-causal temporal smoothing kernels, temporal delay estimation by the mean value may however lead to substantial overestimates compared to *e.g*. the position of the local maximum. To provide more precise characteristics in the case of a uniform distribution of the intermediate temporal scale levels, for which a compact closed form expression is available for the composed kernel

$$\begin{array}{c} h_{composed}\left(t;\;\mu ,K\right)=\frac{t^{K-1}\;e^{-t/\mu }}{\mu^{K}\;\Gamma \left(K\right)} \end{array}$$ (140)

let us differentiate this function

$$\begin{array}{ccc} \partial_{t}\left(h_{composed}\left(t;\;\mu ,K\right)\right) & = & \frac{e^{-\frac{t}{\mu }}\left(\left(K-1\right)\mu -t\right){\left(\frac{t}{\mu }\right)}^{K+1}}{t^{3}\;\Gamma \left(K\right)}\\ \partial_{tt}\left(h_{composed}\left(t;\;\mu ,K\right)\right) & = & \frac{e^{-\frac{t}{\mu }}{\left(\frac{t}{\mu }\right)}^{K}\left(\left(K^{2}-3K+2\right)\mu^{2}-2\left(K-1\right)\mu t+t^{2}\right)}{\mu^{2}\;t^{3}\;\Gamma \left(K\right)} \end{array}$$ (141)

and solve for the positions of the local maximum and the inflection points

$$\begin{array}{ccc} t_{max,uni} & = & \left(K-1\right)\;\mu =\frac{\left(K-1\right)}{\sqrt{K}}\sqrt{\tau }=\frac{2\pi (K-1)\;n}{\sqrt{K}\;\omega } \end{array}$$ (142)

$$\begin{array}{ccc} t_{infl1,uni} & = & \left(K-\sqrt{K-1}-1\right)\mu \\ & = & \frac{\left(K-\sqrt{K-1}-1\right)\sqrt{\tau }}{\sqrt{K}}\\ & = & \frac{2\pi \left(K-\sqrt{K-1}-1\right)n}{\sqrt{K}\;\omega } \end{array}$$ (143)

$$\begin{array}{ccc} t_{infl2,uni} & = & \left(K+\sqrt{K-1}-1\right)\mu \\ & = & \frac{\left(K+\sqrt{K-1}-1\right)\sqrt{\tau }}{\sqrt{K}}\\ & = & \frac{2\pi \left(K+\sqrt{K-1}-1\right)n}{\sqrt{K}\;\omega } \end{array}$$ (144)

Table 3 shows numerical values for the position of the local maximum for both types of time-causal kernels. As can be seen from the table, the temporal response properties are significantly faster for a logarithmic distribution of the intermediate scale levels compared to a uniform distribution, and the difference increases rapidly with *K*. These temporal delay estimates are also significantly shorter than the temporal mean values, in particular for the logarithmic distribution of the intermediate scale levels.

**Table 3:** *Numerical values for the temporal delay of the local maximum in units of* $\sqrt{\tau }$ for time-causal kernels obtained by coupling *K* truncated exponential kernels in cascade in the cases of a uniform distribution of the intermediate temporal scale levels *τ*_*k*_ = *kτ*/*K* or a logarithmic distribution *τ*_*k*_ = *c* ^2(*k* − *K*)^ *τ* with *c* > 1.

Temporal delays from the maxima of time-causal kernels				
*K*	*t*_*max*, *uni*_	*t*_*max*, *log*_ ($c=\sqrt{2}$)	*t*_*max*, *log*_ (*c* = 2^3/4^)	*t*_*max*, *log*_ (*c* = 2)
2	0.707	0.707	0.688	0.640
3	1.154	1.122	1.027	0.909
4	1.500	1.385	1.199	1.014
5	1.789	1.556	1.289	1.060
6	2.041	1.669	1.340	1.083
7	2.268	1.745	1.370	1.095
8	2.475	1.797	1.388	1.100

If we consider a temporal event that occurs as a step function over time (*e.g*. an onset in the magnitude of the spectrogram which is then processed by a second layer of spectro-temporal receptive fields) and if the temporal position of this onset is estimated from the local maximum over time in the first-order temporal derivative response, then the temporal variation in the response over time will be given by shape of the temporal smoothing kernel. The local maximum over time will occur at a time delay equal to the time at which the temporal kernel has its maximum over time. Thus, the position of the maximum over time of the temporal smoothing kernel is highly relevant for quantifying the temporal responses characteristics of time-causal filters.

## Computational implementation

The computational model for auditory receptive fields presented in this paper is based on auditory signals that are assumed to be continuous over time and with frequencies that are also assumed to take values over a continuous frequency domain. When implementing this model on sampled sound signals, the continuous theory must be transferred to discrete time and be restricted to a finite set of discrete frequencies.

In this section we describe how the temporal and spectro-temporal receptive fields can be implemented in terms of corresponding discrete scale-space kernels that possess scale-space properties over discrete temporal and spectro-temporal domains.

### 

#### Discrete temporal scale-space kernels based on recursive filters

Given a temporal signal that has been sampled for some temporal sampling density *ϕ*_0_, the temporal scale *τ* in the continuous model in units of seconds is first transferred to a temporal scale relative to a unit time sampling according to

$$\begin{array}{c} \tau_{sampl}=\phi_{0}^{2}\;\tau \end{array}$$ (145)

where we have here usually used sound signals with *ϕ*_0_ = 44.1 kHz in the experiments. Then, a discrete set of intermediate temporal scale levels is defined according to (38)

$$\begin{array}{c} \tau_{k}=c^{2(k-K)}\tau_{sampl}\;\;\left(1\leq k\leq K\right) \end{array}$$ (146)

or (42)

$$\begin{array}{c} \tau_{k}=\frac{k}{K}\;\tau_{sampl} \end{array}$$ (147)

with the difference between successive scale levels according to (and with *τ*_0_ = 0)

$$\begin{array}{c} \Delta \tau_{k}=\tau_{k}-\tau_{k-1} \end{array}$$ (148)

For implementing the temporal smoothing operation between two such adjacent scale levels, we make use of a *first-order recursive filter*

$$\begin{array}{c} f_{out}\left(t\right)-f_{out}\left(t-1\right)=\frac{1}{1+\mu_{k}}\;\left(f_{in}\left(t\right)-f_{out}\left(t-1\right)\right) \end{array}$$ (149)

with generating function

$$\begin{array}{c} H_{geom}\left(z\right)=\frac{1}{1-\mu \;(z-1)}, \end{array}$$ (150)

which is a time-causal kernel and satisfies discrete scale-space properties of guaranteeing that the number of local extrema or zero-crossings in the signal will not increase (Lindeberg [42]; Lindeberg and Fagerström [44]). Each such filter has temporal mean value *m*_*k*_ = *μ*_*k*_ and temporal variance $\Delta \tau_{k}=\mu_{k}^{2}+\mu_{k}$, and we compute *μ*_*k*_ from Δ*τ*_*k*_ according to

$$\begin{array}{c} \mu_{k}=\frac{\sqrt{1+4\Delta \tau_{k}}-1}{2} \end{array}$$ (151)

By the additive property of variances under convolution for a non-negative kernel, the discrete variances of the discrete temporal scale-space kernels will perfectly match those of the continuous model, whereas the mean values and the temporal delays may be somewhat different. If the temporal scale *τ*_*k*_ is large relative to the temporal sampling density, the discrete model can however be seen as a good approximation also in this respect.

By the time-recursive formulation of this temporal scale-space concept, the computations can be performed based on a compact temporal buffer over time, which contains the temporal scale-space representations at temporal scales *τ*_*k*_ and with no need for storing any additional temporal buffer of what has occurred in the past to perform the corresponding temporal operations.

##### Discrete implementation of Gaussian smoothing

In our model, Gaussian smoothing is used both for smoothing over the spectral domain and non-causal smoothing over the temporal domain. To implement this operation on discrete sampled data, we do first (i) in the case of purely temporal smoothing transform a temporal variance *τ* in units of seconds to a temporal variance relative to a unit sampling density *s*_*sampl*_ according to

$$\begin{array}{c} s_{sampl}=\phi_{0}^{2}\;\tau \end{array}$$ (152)

or (ii) in the case of purely spectral smoothing transform a spectral smoothing scale *σ* in units of semitones to a spectral smoothing scale relative to the logspectral sampling distance Δ*ν* and in units of variance according to

$$\begin{array}{c} s_{sampl}={\left(\frac{\sigma }{\Delta \nu }\right)}^{2} \end{array}$$ (153)

Then, we perform convolution with the *discrete analogue of the Gaussian kernel* (Lindeberg [42])

$$\begin{array}{c} T\left(n;\;s_{sampl}\right)=e^{-s_{sampl}}I_{n}\left(s_{sampl}\right) \end{array}$$ (154)

where *I*_*n*_ denotes the modified Bessel functions of integer order and which corresponds to the solution of the semi-discrete diffusion equation

$$\begin{array}{c} \partial_{s}L\left(n;\;s\right)=\frac{1}{2}\delta_{xx}L=\frac{1}{2}\left(L(n-1;\;s)-2L(n;\;s)+L(n+1;\;s)\right) \end{array}$$ (155)

where *x* denotes the variable over the domain, which can either be time *t* or logarithmic frequency *ν*.

These kernels constitute the natural way to define a scale-space concept for discrete signals corresponding to the Gaussian scale-space over a symmetric domain in the sense of guaranteeing that the number of local extrema or zero-crossings must not increase with scale, while also ensuring a semi-group property

$$\begin{array}{c} T\left(\cdot ;\;s_{1}\right)*T\left(\cdot ;\;s_{2}\right)=T\left(\cdot ;\;s_{1}+s_{2}\right) \end{array}$$ (156)

over the discrete domain which implies that representations at coarser scales can be computed from representations at finer scales using the cascade property (17).

Based on the (exact) relation $\sum_{n=-\infty }^{\infty }T(n;\;s)=1$, we truncate the infinite discrete kernel at the tails

$$\begin{array}{c} \sum_{n=-N}^{N}T\left(n;\;s\right)>1-\varepsilon \end{array}$$ (157)

for some small value of *ɛ* of the order 10^−6^. A coarse estimate of this bound can be obtained by estimating the corresponding tails of the continuous Gaussian kernel

$$\begin{array}{c} 2\int_{x=N}^{\infty }g\left(x;\;s\right)\;dx<\varepsilon \end{array}$$ (158)

using the error function and then adjusting this estimate to match (157).

For points where some part of the kernel stretches outside the domain of available data, we mirror the data at the boundaries, equivalent to solving the diffusion equation with adiabatic boundary conditions—*i.e*. no heat transfer across the boundaries of the domain where data are available.

##### Discrete implementation of spectro-temporal receptive fields

For separable spectro-temporal receptive fields, we implement the spectro-temporal smoothing operation by separable combination of the temporal and spectral scale-space concepts in the appendices “Discrete temporal scale-space kernels based on recursive filters” and “Discrete implementation of Gaussian smoothing”. From this representation, separable spectro-temporal derivative approximations are then computed from *difference operators* of the following types:

$$\begin{array}{c} \delta_{t}=\left(-1,+1\right) \end{array}$$ (159)

$$\begin{array}{c} \delta_{tt}=\left(1,-2,1\right) \end{array}$$ (160)

$$\begin{array}{c} \delta_{v}={\left(-\frac{1}{2},0,+\frac{1}{2}\right)}^{T} \end{array}$$ (161)

$$\begin{array}{c} \delta_{vv}={\left(1,-2,1\right)}^{T} \end{array}$$ (162)

with the difference operators expressed over the appropriate dimensions, here with the implicit convention that time corresponds to the horizontal dimension in an auditory signal or a spectrogram and logarithmic frequency *ν* to the vertical (transposed) dimension.

From the general theory in (Lindeberg [11, 111]) it follows that the scale-space properties for the original zero-order signal will be transferred to such derivative approximations, thereby implying theoretically well-founded implementation of derivative receptive fields.

For non-separable receptive fields corresponding to logarithmic frequencies *ν* that vary with time *t* by glissando *v*, we implement the spectro-temporal smoothing operation by first *warping the spectro-temporal data locally*

$$\begin{array}{c} \nu^{'}=\nu -v\;t \end{array}$$ (163)

using spline interpolation. Then, we apply separable spectro-temporal smoothing in the transformed domain and unwarp the result back to the original domain. Over a continuous domain, such an operation is equivalent to convolution with corresponding glissando-adapted spectro-temporal receptive fields, while being significantly faster in a discrete implementation than corresponding explicit convolution with non-separable receptive fields over two dimensions.

In addition to a transfer of the scale-space properties from the continuous model to the discrete implementation, all the components in this discretization, the discrete Gaussian kernel, the time-recursive filters and the discrete derivative approximations, can be seen as *mathematical approximations of the corresponding continuous counterparts*. Thereby, the behaviour of the discrete implementation will approach the behaviour of the corresponding continuous model as the temporal sampling rate and the sampling rate in the logarithmic frequency domain increase. Choosing appropriate sampling rates in an actual implementation is a trade-off between computational accuracy and computational efficiency.

## References

[1] Aertsen AMHJ, Johannesma PIM (1981) The spectro-temporal receptive field: A functional characterization of auditory neurons. Biological Cybernetics 42: 133–143. 10.1007/BF00336731 7326288

[2] Theunissen FE, Sen K, Doupe AJ (2000) Spectro-temporal receptive fields of nonlinear auditory neurons obtained using natural sounds. The Journal of Neuroscience 20: 2315–2331. 10704507 10.1523/JNEUROSCI.20-06-02315.2000 PMC6772498

[3] Miller LM, Escabi NA, Read HL, Schreiner C (2001) Spectrotemporal receptive fields in the lemniscal auditory thalamus and cortex. Journal of Neurophysiology 87: 516–527. 10.1152/jn.00395.2001 11784767

[4] Fritz J, Shamma S, Elhilali M, Klein D (2003) Rapid task-related plasticity of spectro-temporal receptive fields in primary auditory cortex. Nature Neuroscience 6: 1216–1223. 10.1038/nn1141 14583754

[5] Lindeberg T (2013) A computational theory of visual receptive fields. Biological Cybernetics 107: 589–635. 10.1007/s00422-013-0569-z 24197240 PMC3840297

[6] Iijima T (1962) Observation theory of two-dimensional visual patterns Technical report, Papers of Technical Group on Automata and Automatic Control, IECE, Japan.

[7] Witkin AP (1983) Scale-space filtering In: Proc. 8th Int. Joint Conf. Art. Intell. Karlsruhe, Germany, pp. 1019–1022.

[8] Koenderink JJ (1984) The structure of images. Biological Cybernetics 50: 363–370. 10.1007/BF00336961 6477978

[9] Koenderink JJ, van Doorn AJ (1987) Representation of local geometry in the visual system. Biological Cybernetics 55: 367–375. 10.1007/BF00318371 3567240

[10] Koenderink JJ, van Doorn AJ (1992) Generic neighborhood operators. IEEE Trans Pattern Analysis and Machine Intell 14: 597–605. 10.1109/34.141551

[11] Lindeberg T (1994) Scale-Space Theory in Computer Vision. Springer.

[12] Lindeberg T (1994) Scale-space theory: A basic tool for analysing structures at different scales. Journal of Applied Statistics 21: 225–270. 10.1080/757582976

[13] Lindeberg T (2008) Scale-space In: Wah B, editor, Encyclopedia of Computer Science and Engineering, Hoboken, New Jersey: John Wiley and Sons pp. 2495–2504.

[14] Lindeberg T (2011) Generalized Gaussian scale-space axiomatics comprising linear scale-space, affine scale-space and spatio-temporal scale-space. J of Mathematical Imaging and Vision 40: 36–81. 10.1007/s10851-010-0242-2

[15] Sporring J, Nielsen M, Florack L, Johansen P, editors (1996) Gaussian Scale-Space Theory: Proc. PhD School on Scale-Space Theory Series in Mathematical Imaging and Vision. Copenhagen, Denmark: Springer.

[16] Florack LMJ (1997) Image Structure Series in Mathematical Imaging and Vision. Springer.

[17] ter Haar Romeny B (2003) Front-End Vision and Multi-Scale Image Analysis. Springer.

[18] Hubel DH, Wiesel TN (1959) Receptive fields of single neurones in the cat’s striate cortex. J Physiol 147: 226–238. 10.1113/jphysiol.1959.sp006238 14403679 PMC1363130

[19] Hubel DH, Wiesel TN (1962) Receptive fields, binocular interaction and functional architecture in the cat’s visual cortex. J Physiol 160: 106–154. 10.1113/jphysiol.1962.sp006837 14449617 PMC1359523

[20] Hubel DH, Wiesel TN (2005) Brain and Visual Perception: The Story of a 25-Year Collaboration. Oxford University Press.

[21] DeAngelis GC, Ohzawa I, Freeman RD (1995) Receptive field dynamics in the central visual pathways. Trends in Neuroscience 18: 451–457. 10.1016/0166-2236(95)94496-R 8545912

[22] DeAngelis GC, Anzai A (2004) A modern view of the classical receptive field: Linear and non-linear spatio-temporal processing by V1 neurons In: Chalupa LM, Werner JS, editors, The Visual Neurosciences, MIT Press, volume 1 pp. 704–719.

[23] Conway BR, Livingstone MS (2006) Spatial and temporal properties of cone signals in alert macaque primary visual cortex. Journal of Neuroscience 26: 10826–10846. 10.1523/JNEUROSCI.2091-06.2006 17050721 PMC2963176

[24] Johnson EN, Hawken MJ, Shapley R (2008) The orientation selectivity of color-responsive neurons in Macaque V1. The Journal of Neuroscience 28: 8096–8106. 10.1523/JNEUROSCI.1404-08.2008 18685034 PMC2896204

[25] Young RA (1987) The Gaussian derivative model for spatial vision: I. Retinal mechanisms. Spatial Vision 2: 273–293. 10.1163/156856887X00222 3154952

[26] Young RA, Lesperance RM, Meyer WW (2001) The Gaussian derivative model for spatio-temporal vision: I. Cortical model. Spatial Vision 14: 261–319. 10.1163/156856801753253582 11817740

[27] Lindeberg T (2013) Invariance of visual operations at the level of receptive fields. PLOS ONE 8: e66990 10.1371/journal.pone.0066990 23894283 PMC3716821

[28] Gabor D (1946) Theory of communication. J of the IEE 93: 429–457.

[29] Wolfe PJ, Godsill SJ, Dorfler M (2001) Multi-Gabor dictionaries for audio time-frequency analysis. In: IEEE Workshop on the Applications of Signal Processing to Audio and Acoustics. pp. 43–46.

[30] Lobo AP, Loizou P (2003) Voiced/unvoiced speech discrimination in noise using Gabor atomic decomposition. In: Proc. Acoustics, Speech, and Signal Processing (ICASSP’03). volume 1, pp. 820–823. 10.1109/ICASSP.2003.1198907

[31] Qiu A, Schreiner CE, Escabi MA (2003) Gabor analysis of auditory midbrain receptive fields: Spectro-temporal and binaural composition. Journal of Neurophysiology 90: 456–476. 10.1152/jn.00851.2002 12660353

[32] Wu Q, Zhang L, Shi G (2011) Robust multifactor speech feature extraction based on Gabor analysis. IEEE Trans on Audio, Speech, and Language Processing 19: 927–936. 10.1109/TASL.2010.2070495

[33] Johannesma PIM (1972) The pre-response stimulus ensemble of neurons in the cochlear nucleus In: IPO Symposium on Hearing Theory. Eindhoven, The Netherlands, pp. 58–69.

[34] Patterson RD, Nimmo-Smith I, Holdsworth J, Rice P (1987) An efficient auditory filterbank based on the gammatone function. In: A meeting of the IOC Speech Group on Auditory Modelling at RSRE. volume 2:7.

[35] Hewitt MJ, Meddis R (1994) A computer model of amplitude-modulation sensitivity of single units in the inferior colliculus. The Journal of the Acoustical Society of America 95: 2145–2159. 10.1121/1.408676 8201111

[36] Patterson RD, Allerhand MH, Giguere C (1995) Time-domain modeling of peripheral auditory processing: A modular architecture and a software platform. The Journal of the Acoustical Society of America 98: 1890–1894. 10.1121/1.414456 7593913

[37] Machens CK, Wehr MS, Zador AM (2004) Linearity of cortical receptive fields measures with natural sounds. The Journal of Neuroscience 24: 1089–1100. 10.1523/JNEUROSCI.4445-03.2004 14762127 PMC6793584

[38] Andoni S, Li N, Pollack GD (2007) Spectrotemporal receptive fields in the inferior colliculus revealing selectivity for spectral motion in conspecific vocalizations. Journal of Neuroscience 27: 4882–4893. 10.1523/JNEUROSCI.4342-06.2007 17475796 PMC6672083

[39] Elhilali M, Fritz J, Chi TS, Shamma S (2007) Auditory cortical receptive fields: Stable entities with plastic abilities. The Journal of Neuroscience 27: 10372–10382. 10.1523/JNEUROSCI.1462-07.2007 17898209 PMC6673154

[40] Atencio CA, Schreiner CE (2012) Spectrotemporal processing in spectral tuning modules of cat primary auditory cortex. PLOS ONE 7: e31537 10.1371/journal.pone.0031537 22384036 PMC3288040

[41] Hirschmann II, Widder DV (1955) The Convolution Transform. Princeton, New Jersey: Princeton University Press.

[42] Lindeberg T (1990) Scale-space for discrete signals. IEEE Trans Pattern Analysis and Machine Intell 12: 234–254. 10.1109/34.49051

[43] Lindeberg T (1996) On the axiomatic foundations of linear scale-space In: Sporring J, Nielsen M, Florack L, Johansen P, editors, Gaussian Scale-Space Theory: Proc. PhD School on Scale-Space Theory. Copenhagen, Denmark: Springer, pp. 75–97.

[44] Lindeberg T, Fagerström D (1996) Scale-space with causal time direction In: Proc. European Conf. on Computer Vision (ECCV’96). Cambridge, UK: Springer, volume 1064, pp. 229–240.

[45] Weickert J, Ishikawa S, Imiya A (1999) Linear scale-space has first been proposed in Japan. J of Mathematical Imaging and Vision 10: 237–252. 10.1023/A:1008344623873

[46] Koch C (1999) Biophysics of Computation: Information Processing in Single Neurons. Oxford University Press.

[47] Lindeberg T (1993) Effective scale: A natural unit for measuring scale-space lifetime. IEEE Trans Pattern Analysis and Machine Intell 15: 1068–1074. 10.1109/34.254063

[48] Koenderink JJ (1988) Scale-time. Biological Cybernetics 58: 159–162. 10.1007/BF00364135 3358951

[49] Kleinschmidt M (2002) Methods for capturing spectro-temporal modulations in automatic speech recognition. Acta Acustica united with Acustica 88: 416–422.

[50] Kleinschmidt M, Gelbart D (2002) Improving word accuracy with Gabor feature extraction. In: INTERSPEECH.

[51] van de Boogart CG, Lienhart R (2006) Fast Gabor transformation for processing high quality audio. In: Proc. Acoustics, Speech and Signal Processing (ICASSP’06). volume III, pp. 161–164.

[52] Ezzat T, Bouvrie JV, Poggio T (2007) Spectro-temporal analysis of speech using 2-D Gabor filters. In: INTERSPEECH. pp. 506–509.

[53] Domont X, Heckmann M, Joublin F, Goerick C (2008) Hierarchical spectro-temporal features for robust speech recognition. In: Int. Conf. on Acoustics, Speech and Signal Processing (ICASSP’08). pp. 4417–4420.

[54] He L, Lech M, Maddage N, Allen N (2009) Stress and emotion recognition using log-Gabor filter analysis of speech spectrograms. In: Affective Computing and Intelligent Interaction and Workshops (ACII’09). pp. 1–6.

[55] Heckmann M, Domont X, Joublin F, Goerick C (2011) A hierarchical framework for spectro-temporal feature extraction. Speech Communication 53: 736–752. 10.1016/j.specom.2010.08.006

[56] Schädler MR, Meyer BT, Kollmeier B (2012) Spectro-temporal modulation subspace-spanning filter bank features for robust automatic speech recognition. The Journal of the Acoustical Society of America 131: 4134–4151. 10.1121/1.3699200 22559385

[57] Sameh S, Lachiri Z (2013) Multiclass support vector machines for environmental sounds classification in visual domain based on log-Gabor filters. Int Journal of Speech Technology 16: 203–213. 10.1007/s10772-012-9174-0

[58] Irino T, Patterson RD (1997) A time-domain, level-dependent auditory filter: The gammachirp. The Journal of the Acoustical Society of America 101: 412–419. 10.1121/1.417975

[59] Ambikairajah E, Epps J, Lin L (2001) Wideband speech and audio coding using gammatone filter banks In: IEEE Int. Conf. on Acoustics, Speech, and Signal Processing (ICASSP’01). volume 2, pp. 773–776.

[60] Hohmann V (2002) Frequency analysis and synthesis using a Gammatone filterbank. Acta Acustica United with Acustica 88: 433–442.

[61] van Immerseel L, Peeters S (2003) Digital implementation of linear Gammatone filters: Comparison of design methods. Acoustics Research Letters Online 4: 59–64. 10.1121/1.1573131

[62] Schlute R, Bezrukov L, Wagner H, Ney H (2007) Gammatone features and feature combination for large vocabulary speech recognition. In: IEEE Int. Conf. on Acoustics, Speech and Signal Processing (ICASSP’07). volume IV, pp. 649–652.

[63] Ngamkham W, Sawigun C, Hiseni S, Serdijn WA (2010) Analog complex gammatone filter for cochlear implant channels. In: Proc. 2010 IEEE Int. Symposium on Circuits and Systems (ISCAS). pp. 969–972. 10.1109/ISCAS.2010.5537383

[64] Lindeberg T, Friberg A (2014) Idealized computational models of auditory receptive fields. arXiv preprint arXiv:1404.2037v1 [cs.SD]. 10.1371/journal.pone.0119032 PMC4379182 25822973

[65] Hartmann WM (1996) Pitch, periodicity, and auditory organization. The Journal of the Acoustical Society of America 100: 3491–3502. 10.1121/1.417248 8969472

[66] Moore BCJ (1973) Frequency difference limens for short-duration tones. The Journal of the Acoustical Society of America 54: 610–619. 10.1121/1.1913640 4754385

[67] Johnson DH (1980) The relationship between spike rate and synchrony in responses of auditory-nerve fibers to single tones. The Journal of the Acoustical Society of America 68: 1115–1122. 10.1121/1.384982 7419827

[68] Hartmann WM, McAdams S, Smith BK (1990) Hearing a mistuned harmonic in an otherwise periodic complex tone. The Journal of the Acoustical Society of America 88: 1712–1724. 10.1121/1.400246 2262628

[69] Zwicker E (1961) Subdivision of the audible frequency range into critical bands (frequenzgruppen). The Journal of the Acoustical Society of America 33: 248–248. 10.1121/1.1908630

[70] Moore BCJ (2013) An introduction to the psychology of hearing. Brill Sixth edition.

[71] Palmer SE (1999) Vision Science: Photons to Phenomenology. MIT Press First Edition.

[72] Kandel ER, Schwartz JH, Jessel TM (2000) Principles of Neural Science McGraw-Hill, 4th edition.

[73] Fletcher H (1934) Loudness, pitch and the timbre of musical tones and their relation to the intensity, the frequency and the overtone structure. Journal of the Acoustical Society of America.

[74] Young RW (2005) Terminology for logarithmic frequency units. The Journal of the Acoustical Society of America 11: 134–139. 10.1121/1.1916017

[75] Greenwood DD (1990) A cochlear frequency-position function for several species—29 years later. The Journal of the Acoustical Society of America 87: 2592–2605. 10.1121/1.399052 2373794

[76] Romani GL, Williamson SJ, Kaufman L (1982) Tonotopic organization of the human auditory cortex. Science 216: 1339–1340. 10.1126/science.7079770 7079770

[77] Lindeberg T (1998) Feature detection with automatic scale selection. Int J of Computer Vision 30: 77–116. 10.1023/A:1008045108935

[78] Lindeberg T, Bretzner L (2003) Real-time scale selection in hybrid multi-scale representations In: Griffin L, Lillholm M, editors, Proc. Scale-Space Methods in Computer Vision (Scale-Space’03). Isle of Skye, Scotland: Springer, volume 2695 of *Lecture Notes in Computer Science*, pp. 148–163.

[79] Canny J (1986) A computational approach to edge detection. IEEE Trans Pattern Analysis and Machine Intell 8: 679–698. 10.1109/TPAMI.1986.4767851 21869365

[80] Lindeberg T (1998) Edge detection and ridge detection with automatic scale selection. Int J of Computer Vision 30: 117–154. 10.1023/A:1008045108935

[81] Baer T, Moore BCJ, Gatehouse S (1993) Spectral contrast enhancement of speech in noise for listeners with sensorineural hearing impairment: Effects on intelligibility, quality, and response times. Journal of Rehabilitation Research and Development 30: 49–49. 8263829

[82] Laptev I, Lindeberg T (2004) Velocity-adapted spatio-temporal receptive fields for direct recognition of activities. Image and Vision Computing 22: 105–116. 10.1016/j.imavis.2003.07.002

[83] Lukas BD, Kanade T (1981) An iterative image registration technique with an application to stereo vision. In: Image Understanding Workshop.

[84] Laptev I, Caputo B, Schuldt C, Lindeberg T (2007) Local velocity-adapted motion events for spatio-temporal recognition. Computer Vision and Image Understanding 108: 207–229. 10.1016/j.cviu.2006.11.023

[85] Rodriguez FA, Read HL, Escabi MA (2010) Spectral and temporal modulation tradeoff in the inferior colliculus. Journal of Neurophysiology 103: 887–903. 10.1152/jn.00813.2009 20018831 PMC2822687

[86] Atencio CA, Sharpee TO, Schreiner CE (2012) Receptive field dimensionality increases from the auditory midbrain to cortex. Journal of Neurophysiology 107: 2594–2603. 10.1152/jn.01025.2011 22323634 PMC3362274

[87] Escabi MA, Schreiner CE (2002) Nonlinear spectrotemporal sound analysis by neurons in the auditory midbrain. The Journal of Neuroscience 22: 4114–4131. 12019330 10.1523/JNEUROSCI.22-10-04114.2002 PMC6757655

[88] Marcelja S (1980) Mathematical description of the responses of simple cortical cells. J Optical Society of America 70: 1297–1300. 10.1364/JOSA.70.001297 7463179

[89] Jones J, Palmer L (1987) The two-dimensional spatial structure of simple receptive fields in cat striate cortex. J of Neurophysiology 58: 1187–1211. 10.1152/jn.1987.58.6.1187 3437330

[90] Jones J, Palmer L (1987) An evaluation of the two-dimensional Gabor filter model of simple receptive fields in cat striate cortex. J of Neurophysiology 58: 1233–1258. 10.1152/jn.1987.58.6.1233 3437332

[91] Stork DG, Wilson HR (1990) Do Gabor functions provide appropriate descriptions of visual cortical receptive fields. J Optical Society of America 7: 1362–1373. 10.1364/JOSAA.7.001362 2398445

[92] Meddis R, Lopez-Poveda E, Fay RR, Popper AN (2010) Computational models of the auditory system, volume 35 Springer.

[93] Patterson RD, Moore BCJ (1986) Auditory filters and excitation patterns as representations of frequency resolution In: Moore BCJ, editor, Frequency selectivity in hearing, Academic Press pp. 123–177.

[94] Patterson RD, Robinson K, Holdsworth J, McKeown D, Zhang C, et al (1992) Complex sounds and auditory images. Auditory Physiology and Perception 83: 429–446. 10.1016/B978-0-08-041847-6.50054-X

[95] Chen Z, Hu G, Glasberg BR, Moore BCJ (2011) A new method of calculating auditory excitation patterns and loudness for steady sounds. Hearing Research 282: 204–215. 10.1016/j.heares.2011.09.007 21851853

[96] Lopez-Poveda EA, Meddis R (2001) A human nonlinear cochlear filterbank. The Journal of the Acoustical Society of America 110: 3107–3118. 10.1121/1.1416197 11785812

[97] Patterson RD, Holdsworth J (1996) A functional model of neural activity patterns and auditory images. Advances in Speech, Hearing and Language Processing 3: 547–563.

[98] Bleeck S, Ives T, Patterson RD (2004) Aim-mat: the auditory image model in MATLAB. Acta Acustica United with Acustica 90: 781–787.

[99] Patterson RD, Unoki M, Irino T (2003) Extending the domain of center frequencies for the compressive gammachirp auditory filter. The Journal of the Acoustical Society of America 114: 1529–1542. 10.1121/1.1600720 14514206

[100] Slaney M (1998) Auditory toolbox. Interval Research Corporation, Tech Rep 10.

[101] Ruggero MA (1992) Responses to sound of the basilar membrane of the mammalian cochlea. Current Opinion in Neurobiology 2: 449–456. 10.1016/0959-4388(92)90179-O 1525542 PMC3579517

[102] Chi T, Gao Y, Guyton MC, Ru P, Shamma S (1999) Spectro-temporal modulation transfer functions and speech intelligibility. The Journal of the Acoustical Society of America 106: 2719–2732. 10.1121/1.428100 10573888

[103] Muller M, Ellis DPW, Klapuri A, Richard G (2011) Signal processing for music analysis. IEEE Journal of Selected Topics in Signal Processing 5: 1088–1110. 10.1109/JSTSP.2011.2112333

[104] Brown JC, Puckette MS (1992) An efficient algorithm for the calculation of a constant Q transform. The Journal of the Acoustical Society of America 92: 2698–2701. 10.1121/1.404385

[105] Schörkhuber C, Klapuri A (2010) Constant-Q transform toolbox for music processing In: 7th Sound and Music Computing Conference, Barcelona, Spain, pp. 3–64.

[106] Meyer BT, Kollmeier B (2008) Optimization and evaluation of Gabor feature sets for ASR. In: INTERSPEECH. pp. 906–909.

[107] Eggermont JJ (2011) Context dependence of spectro-temporal receptive fields with implications for neural coding. Hearing Research 271: 123–132. 10.1016/j.heares.2010.01.014 20123121

[108] David SV, Fritz JB, Shamma SA (2012) Task reward structure shapes rapid receptive field plasticity in auditory cortex. Proc National Academy of Sciences 109: 2144–2149. 10.1073/pnas.1117717109 PMC3277538 22308415

[109] Laudanski J, Edeline JM, Huetz C (2012) Differences between spectro-temporal receptive fields derived from artificial and natural stimuli in the auditory cortex. PLOS ONE 7: e50539 10.1371/journal.pone.0050539 23209771 PMC3507792

[110] Pienkowski M, Harrison RV (2005) Tone frequency maps and receptive fields in the developing chincilla auditory cortex. Journal of Neurophysiology 93: 454–466. 10.1152/jn.00569.2004 15342716

[111] Lindeberg T (1993) Discrete derivative approximations with scale-space properties: A basis for low-level feature extraction. J of Mathematical Imaging and Vision 3: 349–376. 10.1007/BF01664794

